# The Acid Phosphatase-Encoding Gene *GmACP1* Contributes to Soybean Tolerance to Low-Phosphorus Stress

**DOI:** 10.1371/journal.pgen.1004061

**Published:** 2014-01-02

**Authors:** Dan Zhang, Haina Song, Hao Cheng, Derong Hao, Hui Wang, Guizhen Kan, Hangxia Jin, Deyue Yu

**Affiliations:** 1 National Center for Soybean Improvement, National Key Laboratory of Crop Genetics and Germplasm Enhancement, Nanjing Agricultural University, Nanjing, China; 2 Department of Agronomy, Henan Agricultural University, Zhengzhou, China; 3 National Center of Plant Gene Research (Shanghai), Shanghai, China; The University of North Carolina at Chapel Hill, United States of America

## Abstract

Phosphorus (P) is essential for all living cells and organisms, and low-P stress is a major factor constraining plant growth and yield worldwide. In plants, P efficiency is a complex quantitative trait involving multiple genes, and the mechanisms underlying P efficiency are largely unknown. Combining linkage analysis, genome-wide and candidate-gene association analyses, and plant transformation, we identified a soybean gene related to P efficiency, determined its favorable haplotypes and developed valuable functional markers. First, six major genomic regions associated with P efficiency were detected by performing genome-wide associations (GWAs) in various environments. A highly significant region located on chromosome 8, *qPE8*, was identified by both GWAs and linkage mapping and explained 41% of the phenotypic variation. Then, a regional mapping study was performed with 40 surrounding markers in 192 diverse soybean accessions. A strongly associated haplotype (*P* = 10^−7^) consisting of the markers Sat_233 and BARC-039899-07603 was identified, and *qPE8* was located in a region of approximately 250 kb, which contained a candidate gene *GmACP1* that encoded an acid phosphatase. *GmACP1* overexpression in soybean hairy roots increased P efficiency by 11–20% relative to the control. A candidate-gene association analysis indicated that six natural *GmACP1* polymorphisms explained 33% of the phenotypic variation. The favorable alleles and haplotypes of *GmACP1* associated with increased transcript expression correlated with higher enzyme activity. The discovery of the optimal haplotype of *GmACP1* will now enable the accurate selection of soybeans with higher P efficiencies and improve our understanding of the molecular mechanisms underlying P efficiency in plants.

## Author Summary

In soybean plants, low-phosphorus (P) stress is more detrimental than other nutrient deficiencies, toxicities or diseases; however, it is difficult to select soybean varieties with high P efficiencies based on phenotypes. Although QTL map-based cloning is a powerful method, it is time-consuming, and the QTLs underlying P efficiency in soybeans have not been identified. Combining linkage and association analyses, we identified a highly significant region on chromosome 8, *qPE8*, which is associated with soybean P efficiency. Gene expression and plant transformation experiments indicated that the gene *GmACP1* within *qPE8* affects P efficiency. A haplotype survey of *GmACP1* identified 10 haplotypes that explained 33% of the variation in P efficiency. The discovery of the optimal haplotype of *GmACP1* will improve the accuracy of selecting soybeans with higher P efficiencies and increase our understanding of the molecular mechanisms underlying P efficiency in plants.

## Introduction

Phosphorus (P) is essential for all living cells and organisms. P occurs in complex DNA and RNA structures that contain and translate genetic information and control all living processes in plants and animals. Animals need to obtain an adequate supply of P from their food [1]. As for plants, P is one of the most essential mineral nutrients required for growth and development [2]. However, most unmanured soils do not contain sufficient readily available P to meet the high demands of crops, particularly during certain periods of the growing cycle. Therefore, fertilizers containing P must be supplied. In recent years, the annual global consumption of phosphates was more than 50 million tons [3]. However, excess P application is problematic because the excess dissolution of phosphates contaminates water sources [4], [5]. Furthermore, most of the annually fertilized phosphates are fixed in the soil in organic forms by adsorption, sedimentation and transformation. Consequently, 50–80% of the total P found in soil exists as organic phosphates, which are unavailable to plants in the absence of mineralization [6]. Therefore, agricultural soil is a large ‘potential P pool’ that must be developed and used. Furthermore, the global reserve of rock phosphate is a non-renewable resource. A recent study conducted by the International Fertilizer Development Center (IFDC) concluded that at the current extraction rates, the global commercial phosphate reserves will be depleted in approximately 300–400 years [7]. Therefore, the development of plants that can efficiently utilize endogenous and added P is a sustainable, economic approach for plant production.

In response to persistent P deficiency, plants have developed many adaptive mechanisms to enhance the availability and uptake of P, including modifications in root architecture, increased activities of internal and extracellular acid phosphatases, greater exudation of small molecular organic acids, and symbiosis with mycorrhizal fungi [2]. Hundreds of genes associated with the plant P metabolic pathway have been identified [2], but few of these have been applied in crop breeding programs, perhaps because most of the identified genes were cloned by reverse genetics methods from a few model plants [6], [8]–[10], while few were identified by forward genetics methods [11]. For example, tremendous efforts have been made to dissect the genetic basis of soybean P efficiency by assessing factors such as biomass [12], root architecture [13], P concentration, acid phosphatase activity [14] and flower/pod abscission rates [15]. These studies, which were based on genetic analyses in segregating populations, identified several QTLs that likely control P efficiency. However, no QTLs associated with soybean P efficiency have been cloned. The reasons for these limited results may include the following: (a) the QTLs associated with P efficiency in soybean are generally localized to large chromosomal regions (10–20 centimorgans), (b) the effects of the QTLs are minor and often represent only a small fraction of the phenotypically relevant variation, and (c) QTLs are complex, and their evaluation requires information regarding germplasm diversity. Therefore, many challenging questions remain unanswered. For example, how can we identify the candidate P efficiency genes in these QTLs? Is P efficiency modulated by genetic variation of the candidate genes? What are the most favorable alleles and haplotypes useful for the breeding of high P efficiency? These questions reveal the highly challenging nature of the genetic dissection of P efficiency in soybean and other plants.

Although QTL linkage mapping provides useful information on genetic loci, it is typically difficult to isolate candidate genes based on a single QTL mapping experiment. Furthermore, the genes identified by this method are restricted to those that segregate in the considered cross [16]. Genome-wide associations (GWAs) overcome this limitation and have recently been successfully applied in studies of *Arabidopsis* [17], rice [18], [19], maize [20] and other plants [21], [22]. This method can provide increased power for the localization of QTLs because of the higher recombination rates between markers and QTL alleles in randomly mating populations [23]–[25]. However, GWAs also have limitations because they could generate false positives as a result of population structure. Although population structure can be controlled by statistical methods [13], [17], [18], the complementary use of family-based linkage analyses in controlled crosses is also an option [26], [27]. The complementarity of GWAs and classical linkage analyses has been well demonstrated by studies of *Arabidopsis* flowering time [16] and rice Al tolerance [28]. In addition, because P efficiency is a typical quantitative trait controlled by multiple genes [29] and the molecular mechanism underlying soybean P efficiency is poorly understood, a candidate-gene association analysis could be an effective means to functionally characterize target genes. This strategy to study complex quantitative trait genes has been successfully applied for many plant traits such as maize flowering time [23], carotenoid content [30], architecture of floral branch systems [31] and rice seed shattering-loss [32].

Soybean is a highly important crop. Low P availability is the most significant soybean production constraint and is more problematic than other nutrient deficiencies, toxicities or diseases [33]. Because soybean is a model legume plant, the characterization of P efficiency related genes and the mechanisms responding to low-P stress in soybean would eventually facilitate P efficiency studies in other legumes and plants. We aimed to clone the soybean candidate gene for P efficiency, elucidate its effects, determine its favorable haplotypes and develop valuable functional markers. Therefore, we performed a series of experiments including linkage mapping, genome-wide association mapping, candidate-gene association mapping, gene expression and plant transformation. These studies revealed that a soybean acid phosphatase-encoding gene, *GmACP1*, located within the major QTL *qPE8*, is associated with P efficiency, and its genetic variation modulates P efficiency related traits in soybeans.

## Results

### Significant variation in P efficiency among soybean germplasms

To determine the genetic variation of P efficiency in soybean plants, four P-efficiency-related traits were determined using 152 soybean recombination inbred lines (RILs) (Table 1) and 192 soybean accessions (Table 2). These traits included plant height (PHt), acid phosphatase activity (APA), leaf P concentration (PC) and 100-seed weight (100-SW) under low-P (−P, soil available P<5 mg kg^−1^) and high-P (+P, soil available P>20 mg kg^−1^) conditions; the relative values of the traits −P/+P are denoted as RPH, RAPA, RPC and 100-RSW, respectively. As shown in Table 1, the phenotypic RIL values ranged from 0.12–0.62 mg g^−1^ for PC, 0.13–0.58 mg g^−1^ for the pod P concentration (PPC) and 0.70–3.01 µmol ρ-NP min^−1^ mg protein^−1^ for APA in the low-P condition. The transgressive segregation of P-efficiency-related traits was obvious, and the phenotypic variation was significantly affected by the genotypes and treatments. In addition, analysis of variance (ANOVA) indicated that the phenotypic APA variation between the two parents (Bogao and Nannong 94–156) was significant (*P* = 0.01) in different P conditions (Table 1). The mean APA values for the individual accessions in the natural population ranged from 1.34–2.12 µmol ρ-NP min^−1^ mg protein^−1^, and the mean PC values ranged from 0.07–1.84 mg g^−1^. Among the lines of diverse soybean accessions, the PC levels reached 1.84 mg g^−1^; however, one soybean accession had a PC of only 0.07 mg g^−1^ (Table 2). Overall, the soybean plants clearly exhibited considerable natural variation in the traits related to P efficiency and displayed very high genetic diversity.

**Table 1:** Descriptive statistical results for traits related to phosphorus (P) efficiency in soybean recombinant inbred lines (RILs) and their parents in experiments conducted in 2006 and 2007.

			Population								Parents	
Trait	Treatment	Year	Mean±SD	Range	Skew	*h^2^* (%)^a^	R^b^	G^c^	T^d^	Y^e^	Bogao	Nannong 94–156
PC	−P	2006	0.30±0.08	0.12–0.53	0.94	57.1	ns	s	s	ns	0.31	0.41
		2007	0.31±0.09	0.12–0.62	0.12						0.31	0.45
	+P	2006	0.40±0.09	0.20–0.77	0.69	41.3					0.41	0.45
		2007	0.40±0.08	0.20–0.72	0.46						0.43	0.57
PPC	−P	2006	0.33±0.07	0.15–0.58	0.54	57.6	ns	s	s	ns	0.38	0.41
		2007	0.30±0.08	0.13–0.56	0.33						0.39	0.46
	+P	2006	0.41±0.08	0.26–0.66	0.72	50.6					0.49	0.55
		2007	0.38±0.08	0.22–0.65	0.57						0.51	0.54
APA	−P	2006	1.41±0.32	0.70–3.01	0.81	31.6	ns	s	s	s	1.23	1.52
		2007	1.45±0.40	0.75–2.80	0.98						1.31	1.42
	+P	2006	1.30±0.29	0.53–2.08	−0.02	38.9					1.01	1.22
		2007	1.02±0.37	0.45–2.27	0.84						1.12	1.13
PHt	−P	2006	51.62±14.00	30.00–88.00	0.25	73.4	ns	s	s	s	60.38	33.5
		2007	57.1±14.08	25.83–97.33	−0.24						61.33	35.2
	+P	2006	56.79±14.24	23.75–83.75	0.24	84.5					65.75	37.25
		2007	64.62±13.24	24.33–89.05	−0.26						69.56	40.2
100-SW	−P	2006	17.1±2.55	7.00–23.00	0.02	45.5	ns	s	s	ns	13.36	14.51
		2007	15.99±2.65	8.72–22.45	−0.02						12.34	13.5
	+P	2006	18.46±2.31	12.98–27.77	0.74	62.5					17.28	19.12
		2007	17.67±2.64	11.76–26.60	0.53						16.45	17.56

s: significant difference at *P* = 0.01; ns: not significant; PC: P concentration in the plant (mg g^−1^); PPC: P concentration in the pod at the seed filling stage (mg g^−1^); APA: acid phosphatase activity (µmol min^−1^ mg protein^−1^); PHt: plant height (cm); 100-SW: 100-seed weight (g).

^a^ heritability;

^b^ replication;

^c^ genotype;

^d^ treatment;

^e^ year.

**Table 2:** Descriptive statistical results for traits related to phosphorus (P) efficiency in 192 soybean accessions in experiments conducted in 2008 and 2009.

Trait	Treatment	Year/Site	Mean±SD	Range	Skew	R^a^	G^b^	T^c^	Y^d^
PC	−P	2008HN	0.28±0.08	0.14–0.52	0.14	ns	s	s	s
		2008NJ	0.28±0.10	0.14–1.00	1.5				
		2009NJ	0.50±0.31	0.07–1.53	0.97				
	+P	2008HN	0.35±0.07	0.16–0.67	0.37				
		2008NJ	0.40±0.12	0.13–1.00	0.74				
		2009NJ	0.82±0.45	0.15–1.84	0.37				
APA	−P	2008HN	2.12±0.66	0.12–6.47	0.99	ns	s	s	ns
		2008NJ	1.80±0.69	0.49–7.25	0.86				
		2009NJ	1.96±0.60	0.55–6.15	1.2				
	+P	2008HN	1.61±0.62	0.10–2.62	−0.35				
		2008NJ	1.34±0.46	0.39–3.80	0.44				
		2009NJ	1.54±0.38	0.18–3.77	0.73				

s: significant difference at *P* = 0.01; ns: not significant; PC: P concentration in the plant (mg g^−1^); APA: acid phosphatase activity (µmol min^−1^ mg protein^−1^); HN and NJ: field experiments in Henan and Nanjing, China, respectively.

^a^ replication;

^b^ genotype;

^c^ treatment;

^d^ year.

### P efficiency related QTL linkage mapping in the RIL population

Several P-efficiency-related QTLs have been identified in our previous works [14], [15], most of which focused on P efficiency at the soybean seedling stage. However, 90% of soybean P absorption occurs during the reproductive stage [33], [34]. Therefore, studying the P-efficiency-related traits during the reproductive stage is expected to be better suited for identifying the QTL associated with P efficiency. In this study, we initially aimed to identify the QTLs related to P efficiency during the soybean reproductive stage using 152 RILs in various environments. We used the relative values RPH, RPC, RPPC, RAPA and 100-RSW as indices to assess P efficiency in soybeans. Three primary QTL were found to be associated with P efficiency which were located on three soybean chromosomes (8, 14 and 18) (Table 3). Of the three loci, a stable QTL (for RAPA, RPPC and RPC) termed *qPE8* (QTL for P efficiency related traits on chromosome 8) was identified; this QTL encompasses a 6.3-Mb region and explained up to 41% of the phenotypic variation (Table 3, Figure S1). This locus was consistent with previously identified QTL associated with P efficiency in soybean [14], [15].

**Table 3:** QTL analysis for traits related to phosphorus (P) efficiency in experiments conducted in 2006 and 2007 using 152 soybean recombinant inbred lines (RILs).

Trait	Year	Chr.	Marker interval	Confidence Interval	LOD	ADD.	*R^2^* (%)
RPC	2006	Chr.8	Satt089-Sat-310	155.8–161.7	9.8	0.17	41.05
		Chr.13	Sat_262-Satt030	30.0–53.3	2.8	−0.08	7.72
	2007	Chr.8	Satt089-Sat-310	156.0–163.4	13.4	0.17	37.74
RPPC	2006	Chr.8	Satt089-Sat-310	154.9–162.1	11.9	0.11	31.07
	2007	Chr.5	Satt276-Sat_368	27.8–35.1	2.7	−0.05	5.67
		Chr.8	Satt089-Sat-310	150.7–154.3	8.5	0.11	22.41
RAPA	2006	Chr.8	Satt089-Sat-310	150.9–160.2	6.8	0.2	18.05
	2007	Chr.8	Satt089-Sat-310	141.0–160.2	3.5	0.14	19.02
RPHt	2006	Chr.14	Sat_424-Satt063	37.5–40.5	9.1	−0.03	21.28
		Chr.1	Satt436-Sat_343	79.7–91.0	3.1	−0.02	8.2
	2007	Chr.14	Sat_424-Satt063	37.6–40.6	10.2	−0.04	23.2
		Chr.1	Satt436-Sat_346	88.0–92.7	3.2	−0.03	6.75
		Chr.19	Sat_191-Satt313	201.1–214.3	3.2	0.02	7.05
100-RSW	2006	Chr.18	Sat_185-Satt012	63.8–65.8	13.5	0.08	26.06
		Chr.18	Sat_352-Satt138	106.60–112.2	4.6	0.04	7.54
	2007	Chr.18	Sat_185-Satt012	63.6–67.4	4.9	0.13	14.18

ADD: additive effect; *R^2^*: the contribution ratio of QTL effect; RPC: relative P concentration in the plant at two P conditions; RPPC: relative P concentration in the pod at the filling stage at two P conditions; RAPA: relative acid phosphatase activity at two P conditions; RPHt: relative plant height at two P conditions; 100-RSW: relative value of 100-seed weight at two P conditions.

### GWAs for P efficiency related traits in a natural population

**Figure 1:**
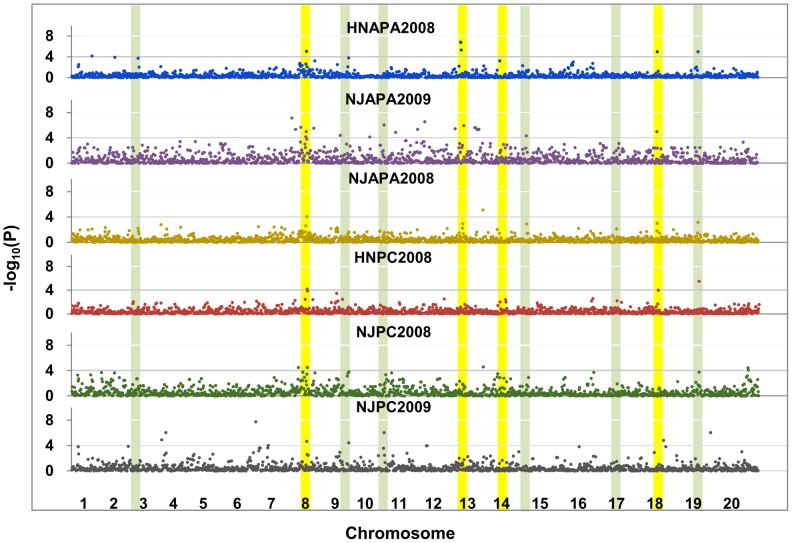
Genome wide associations (GWAs) of phosphorus (P) efficiency across traits, sites and years in 192 soybean accessions. GWAs across traits, sites and years (RAPA-HN/NJ2008 denotes the relative acid phosphatase activity under −P and +P obtained in Henan/Nanjing in 2008, RPC-HN/NJ2008 denotes the relative P concentration under −P and +P obtained in Henan/Nanjing in 2008). Chromosomes listed in the red font denote the six major significant SNP clusters associated with P efficiency identified by the GWAs. The color bands indicate the 10 major bi-parental QTL clusters identified in previous reports (green) or in the current study (yellow) by linkage mapping.

To overcome the limitations of linkage analysis, we conducted GWAs to identify the loci associated with P efficiency using 1,536 single nucleotide polymorphisms (SNPs) [35] and the relative phenotypic values at several sites and over several years as P efficiency phenotypes generated in 192 soybean accessions. GWAs were conducted across all 192 genotypes using SNPs with minor allele frequencies (MAF)>0.05. In addition, the GWAs were separately associated with the P efficiency related phenotype, APA and PC across sites and years (Figure 1). A mixed linear model (MLM), which controlled for the complex population structure and pedigree relationships, was used in each analysis to correct for the confounding effects of the subpopulation structure and relatedness between individuals. The relative performance of the MLM in all traits was evaluated to control false positives in the studied population, as shown in the quantile-quantile (QQ) plots (Figure S2). The smaller deviation of the observed *P* value from the expected value indicated that the MLM was suitable for the control of type I errors. Seventy-four significant SNPs associated with P efficiency were identified and organized by the GWAs into six major clusters on chromosomes 8, 9, 11, 13, 18 and 19 across various environments (Figure 1).

**Figure 2:**
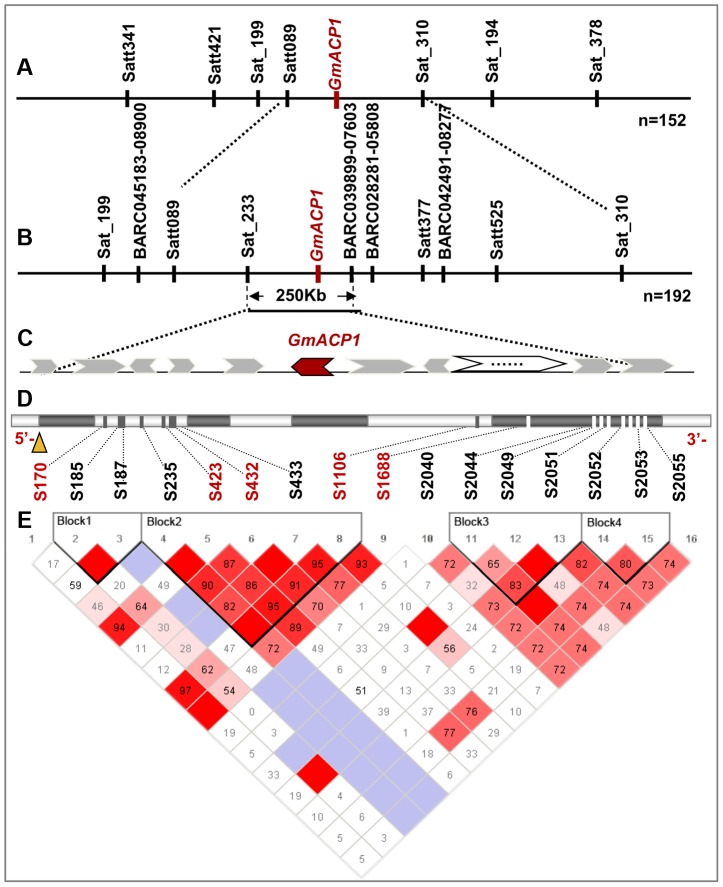
Fine mapping and positional cloning of *GmACP1*. (A) A phosphorus efficiency quantitative trait locus (QTL) *qPE8* was mapped to the interval between the markers Satt089 and Sat_310 on soybean chromosome 8 using 152 recombinant inbred lines (RILs). (B) This QTL was further delimited to an approximately 250 kb region on chromosome 8 using a natural population composed of the 192 accessions. (C) The arrow indicates the predicted gene between the markers Sat_233 and BARC-039899-07603, including the five candidate genes *Glyma08g20700*, Calcineurin B; *Glyma08g20710*, Phospholipase D; *Glyma08g20800* and *Glyma08g20820*, Putative Phosphatase; and *Glyma08g20830*, Protein Phosphatase. (D) The *GmACP1* gene model showing the allelic variation (frequency >5%) of the *GmACP1* sequence. (E) Linkage disequilibrium (LD) plot between *GmACP1* SNPs. The physical position of each SNP is shown above the plot. The magnitude of LD indexed by the D′ statistic is also shown. Red squares without numbers indicate complete LD (D′ = 1, *P*<0.01). D′ values are shown in the squares for values <1.0. Pale blue squares indicate D′ = 1 but inter-marker *P*≥0.01.

Next, the SNPs identified by the GWAs were compared to the positions of QTL regions identified by linkage mapping in this study and previous reports (Figure 1). First, the results indicated that the GWAs identified more phenotype-genotype associations and provided higher resolution; however, linkage QTL mapping identified rare specific alleles that were undetectable by the GWAs. Second, most of the highly significant associated regions identified by the GWAs were also identified and validated by linkage QTL mapping (Figure 1). Finally, a highly significant SNP cluster (including four SNPs, *P* = 7.5×10^−8^) on chromosome 8, co-localized to *qPE8*, was found to be flanked by Satt089 and Sat_310. In addition, previous studies have shown that this locus is associated with P efficiency at the soybean seedling stage [9], [10]. Therefore, we analyzed *qPE8* to identify the P-efficiency-related genes located within this region. At this stage of the study, however, it was not possible to discriminate among the candidate genes for P efficiency in this region. Therefore, we decided to investigate the region between Satt089 and Sat_310 (Figure 2A).

### Regional association mapping to refine the *qPE8* region within 250 kb

To refine the major QTL region *qPE8*, which was identified by both association and linkage mapping and shown to stably affect soybean P efficiency across various environments, we performed a regional association mapping analysis with 18 SSR markers and 22 SNP markers (an average of one marker per 0.15 Mb) in a population of 192 soybean accessions (Figure 2B). The regional scan of the association analysis revealed that six markers (BARC-045183-08900, Satt089, Sat_233, BARC-039899-07603, BARC-028281-05808 and Satt377) were significantly associated with the RAPA in the *qPE8* genomic region (Table 4). Of these, Sat_233 and BARC-039899-07603 were closely associated with the RAPA (*P* = 2.0×10^−6^ and *P* = 6.7×10^−5^, respectively). In addition, the haplotype between Sat_233 and BARC-039899-07603 explained more of the phenotypic variation (27.2%) than any other combination of markers. These results suggested that the target gene was located in a region of approximately 250 kb between Sat_233 and BARC-039899-07603 (Figure 2C).

**Table 4:** Markers associated with the relative acid phosphatase activity (RAPA) in two phosphorus conditions and their phenotypic variations.

Marker	Position (Mb)	*P* a	*R^2^* b
BARC-045183-08900	15.13 Mb	3.4×10^−2^	2.70%
satt089	15.30 Mb	1.6×10^−3^	7.90%
Sat_233	15.56 Mb	2.02×10^−6^	14.60%
BARC-039899-07603	15.82 Mb	6.7×10^−5^	11.20%
BARC-028281-05808	15.93 Mb	1.04×10^−4^	10.80%
Satt377	16.43 Mb	0.13	2.90%

^a^ *P* value from the ANOVA analysis of relative acid phosphatase activity in 2009 (Nanjing),

b *R^2^* showing the percentage phenotypic variation obtained from the ANOVA.

### *GmACP1* is the candidate gene for the QTL *qPE8*

A comprehensive analysis of the region between Sat_233 and BARC-039899-07603 predicted 28 annotated genes (Table S1). Five of the 28 genes (*Glyma08g20700, 20710, 20800, 20820* and *20830*) were considered candidate genes for P efficiency or plant stress following BLASTP queries of the protein database (http://www.ebi.ac.uk/Tools/sss/ncbiblast/) and synteny analyses between soybean and other dicotyledonous plants. The candidate genes included *Glyma08g20700*, which encodes calcineurin B, whose *Arabidopsis* homolog responds to abiotic stress [36], and *Glyma08g20710*, which encodes phospholipase D, whose *Arabidopsis* homolog responds to phosphate starvation [37]. The candidate gene *Glyma08g20830* encodes a protein phosphatase whose yeast homolog is essential for eliciting adequate cellular responses to stress [38]. The candidate genes *Glyma08g20800* and *Glyma08g20820* encode putative phosphatases, and their homologs in the common bean and tomato are strongly induced during P starvation [39], [40].

To assess the responses of these five putative candidate genes to low-P stress in soybean, a quantitative real-time PCR (qRT-PCR) analysis was performed using two representative accessions. The candidate gene *Glyma08g20820*, which encodes an acid phosphatase (termed GmACP1), was dramatically upregulated during low-P stress (Figure S3). However, the expression of the remaining four genes did not change significantly in response to low-P stress. *GmACP1* is 2,283 bp long and encodes a 272-amino acid polypeptide. The deduced amino acid sequence of GmACP1 is 86% identical to a common bean acid phosphatase (*PvPS2*) and 70% identical to a tomato acid phosphatase (*LePS2*) (Figure S4). An alignment of the amino acid sequences of GmACP1 and other plant phosphatases revealed two highly conserved motifs, namely, motif 1 “DFDXT” and motif 2 “GDGXXD”, which are members of the haloacid dehalogenase (HAD) and DDDD (4-aspartate) enzyme superfamilies, respectively (Figure S4). These enzymes catalyze various hydrolytic and phosphotransferase reactions [41], [42]. Furthermore, two conserved aspartic acid residues were identified in the DFDXT motif; the first Asp might be transiently phosphorylated during the P-transfer reaction [43]. The second conserved motif, GDGXXD, is generally observed in phosphatases rather than phosphomutases [42], [43]. These results indicate that *GmACP1* may encode a putative phosphatase in soybean.

**Figure 3:**
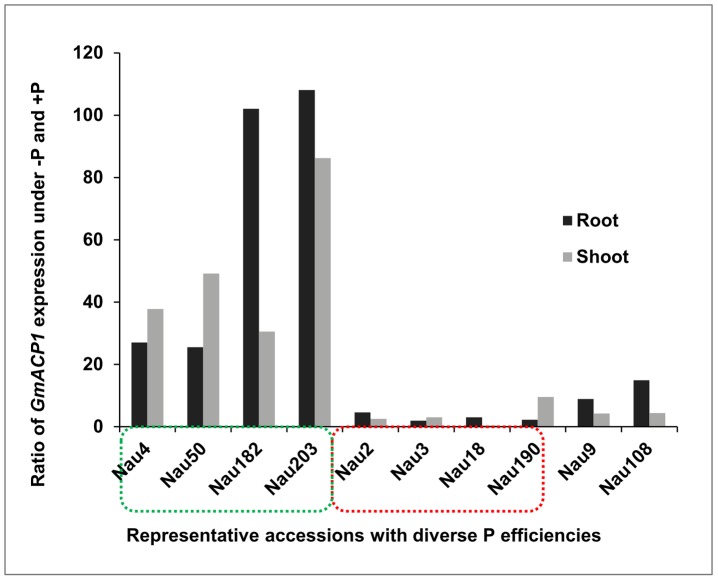
Quantitative real-time PCR of *GmACP1* in ten representative accessions with diverse phosphorus (P) efficiencies. The Y-axis denotes the ratio of *GmACP1* expression under low P (−P) and high P (+P, control) conditions after seven days. The levels of *GmACP1* in the accessions with high P efficiency marked by the green box (Nau4, Nannong 94–156, the high P efficiency parent of the RILs; Nau50; Nau182 and Nau203) were increased by 26- to 110-fold; those in the accessions with low P efficiency marked by the red box (Nau3, Bogao, the low P efficiency parent of the RILs; Nau2; Nau18 and Nau190) were increased by 2- to 9-fold. Nau9 and Nau108 are representative accessions with moderate P efficiencies.

To further confirm the candidate gene, we analyzed *GmACP1* expression by performing qRT-PCR on different soybean plant tissues. *GmACP1* was expressed at high levels in the roots, shoots and leaves and low levels in the flowers, but it was undetectable in the pods (data not shown). qRT-PCR was then conducted on the roots and shoots of eight representative soybean accessions, including the two parents used for linkage mapping. *GmACP1* expression was increased by 26- to 110-fold in the accessions with high P efficiency (such as Nau 4); however, the expression was increased by only 2- to 9-fold in the accessions with low P efficiency (such as Nau 3) (Figure 3).

**Figure 4:**
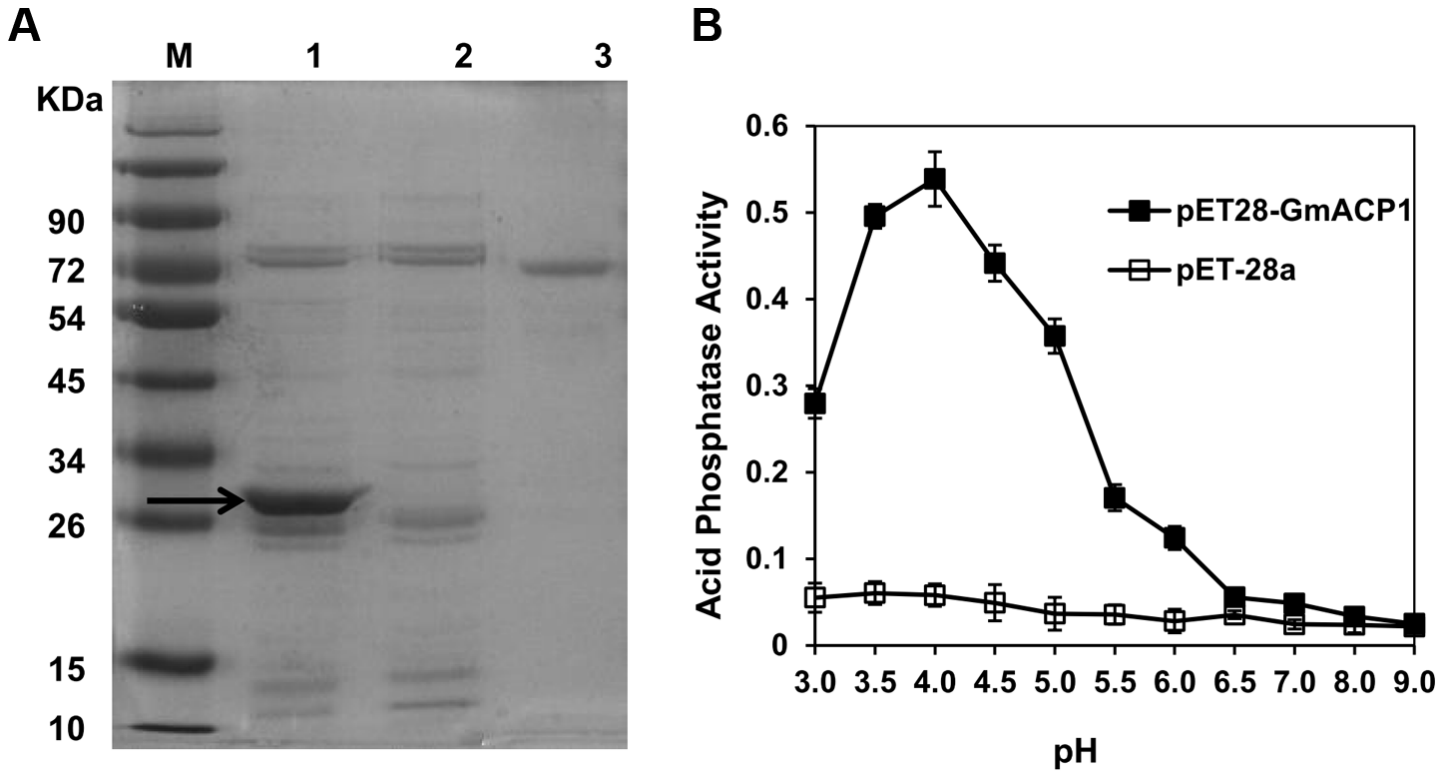
Expression of *GmACP1* in *E. coli*. (A) SDS-PAGE of the GmACP1 recombinant protein. The *GmACP1* coding sequence was cloned into a *pET28a* vector and introduced into BL21 cells. The cells were induced with IPTG to produce the recombinant protein. The bacterial proteins were separated on a 10% (w/v) SDS-PAGE gel. M, protein marker; 1, Ni affinity column-purified recombinant GmACP1 protein (BL21 carrying the *GmACP1* coding sequence in a *pET28a* vector); 2, Ni affinity column-purified control protein (BL21 carrying the *pET28a* vector); 3, Bovine serum albumin (BSA) (1 mg/mL). The arrow indicates the 31.8-kDa GmACP1 protein. (B) Acid phosphatase activity of the recombinant GmACP1 protein at different pH values. The synthetic substrate *ρ*-nitrophenol phosphate was used to quantify the activity of the column-purified GmACP1 protein. The assay was performed using bacterial extracts containing the vector alone (*pET28a*) as a control or the recombinant plasmid (*pET28a-GmACP1*). The error bars represent the SDs of all of the tested samples.

The *GmACP1* coding sequence was expressed in *Escherichia coli* (BL21). The bacteria expressed high levels of GmACP1 following induction with isopropylthio-*β*-galactoside (IPTG), and the protein was detected in the soluble fraction. The expressed protein was resolved as a 31.8-kDa peptide on an SDS-PAGE gel (Figure 4A). The recombinant protein had a low but significant phosphatase activity relative to the control. The phosphatase activity had an optimal pH of 4.0 (Figure 4B), suggesting that *GmACP1* encodes an acid phosphatase and is the candidate gene in the QTL *qPE8*.

### Soybean hairy root transformation confirms the function of *GmACP1*

**Figure 5:**
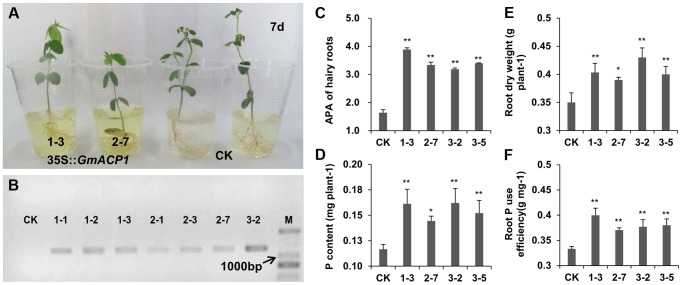
Phenotypes of hairy roots overexpressing *GmACP1* and control hairy roots (CK) cultured by hydroponics and supplied with 0.5 mM phytate. (A) *In situ* staining for the activity of acid phosphatase at day 7. The yellow color indicates the enzyme activity in roots. (B) The PCR of hairy root DNA using the primers (35S-F+*GmACP1*-R) to amplify a 1,343-bp fragment. M, Marker; CK, soybean hairy roots transformed with the control vector pMDC83; 1 to 7, individual plants transformed with the binary vector *GmACP1*-pMDC83. (C–F) The effects of hairy roots overexpressing *GmACP1* on soybean root dry weight, P concentration and root P use efficiency (ratio of root dry weight to P concentration). The comparisons were performed using ANOVA. * and ** denote significant differences at P = 0.05 and 0.01, respectively.

To elucidate the function of *GmACP1*, a *GmACP1*-pMDC83 overexpression vector was constructed and introduced into soybean roots via *Agrobacterium rhizogenes*-mediated transformation [44]. The transgenic hairy roots were verified by PCR amplification (Figure 5B). After 20 days of growth on vermiculite, the transgenic hairy roots were transferred into B&D solution (Broughton and Dilworth, 1971) supplemented with *ρ*-nitrophenyl phosphate (*ρ*-NPP) for seven days. The yellow color generated by the secreted acid phosphatase was more intense in the transgenic hairy roots overexpressing *GmACP1* than in the hairy roots transformed with the control vector (Figure 5A). Furthermore, the control plants but not the composite transgenic plants developed the symptoms of P deficiency, such as curled leaf edges and deep green leaves. The transgenic soybean hairy roots exhibited a 2.3-fold increase in APA and 11.2–20.0% more efficient P usage by the roots relative to the controls. The transgenic soybean hairy roots also exhibited improved P efficiencies with 11.4–22.8% increases in root dry weight and 23.8–39.0% increases in root P concentration, compared with the controls (Figure 5C–F).

### *GmACP1* polymorphisms are associated with soybean P efficiency related traits

Sequencing analysis indicated that polymorphisms (including single-base changes and indels) existed in the exons and introns of the *GmACP1* genes from the 192 soybean accessions, including the two parents used for the linkage mapping analysis. We identified and filtered out 16 SNPs (Figure 2D) for the subsequent association analyses. Furthermore, we observed that these 16 SNPs exhibited strong linkage disequilibrium (LD) (Figure S5) and could form four LD blocks (Figure 2E). Because *GmACP1* was upregulated in response to low-P stress, and overexpression of *GmACP1* increased the APA and P efficiency in soybean roots, we evaluated whether *GmACP1* polymorphisms were correlated with the variation of P efficiency related traits; we observed that *GmACP1* polymorphisms between the two parents were significantly associated with APA variation (Figure S6). As shown in Table 5, gene associations in the 192 soybean accessions confirmed that *GmACP1* plays a key role in regulating the APA and PC. Six probable causative sites, including Indel170, Indel423, Site 432 (S432) and S433 in intron 1, S1106 in intron 3 and S1688 in exon 4, were significantly associated with variations in P efficiency related traits after analysis by Tassel version 4.0 [45] (Table 5). Indel170 was significantly correlated with the RAPA, accounting for 32% of the phenotypic variation (*P* = 1.84×10^−41^), which was estimated by multiple linear regression (MLR) using SAS version 9.0 (SAS Institute Inc., Cary, NC, USA), and RPC accounted for 23% of the phenotypic variation (*P* = 5.32×10^−31^, MLR). The accuracy of the algorithm (MLR) was estimated using 5-fold cross validation, and the frequency distributions of R^2^ based on the 5-fold cross validation are shown in Figure S7. A single-base transversion at S1688 resulted in the substitution of tyrosine with phenylalanine, which explained 22% of the variation (*P* = 5.35×10^−31^, MLR).

**Table 5:** *GmACP1* polymorphisms associated with phosphorus (P) efficiency traits in diverse soybean accessions.

			Environment (Observation no. RAPA/RPC)					
Trait	Polymorphic site^a^	Allele in series^b^	2008NJ^c^ (171/184)	2008HN (170/171)	2009NJ (184/157)	*R^2^* d	*P* e	Fold change in haplotypes^f^
RAPA	InDel170	1/2/3/**4/**5	2.54×10^−13^	1.93×10^−9^	1.85×10^−5^	32%	1.84×10^−41^	2.6
	InDel423	1/2/**0**	1.96×10^−12^	4.77×10^−9^	5.28×10^−9^	23%	5.32×10^−31^	1.8
	S432	A/**T**	9.65×10^−3^	ns	6.65×10^−4^	4%	6.16×10^−5^	1.3
	S433	A/**T**	9.05×10^−3^	ns	4.23×10^−4^	4%	1.26×10^−4^	1.2
	S1106	G/**A**	1.07×10^−12^	3.65×10^−9^	1.10×10^−7^	23%	5.35×10^−30^	2.1
	S1688	A/**T**	3.38×10^−13^	1.78×10^−8^	1.82×10^−7^	22%	2.47×10^−31^	2.2
RPC	InDel170	1/2/3/**4**/5	4.83×10^−10^	1.76×10^−7^	1.33×10^−6^	19%	4.17×10^−21^	2.4
	InDel423	A/**1**	2.07×10^−7^	1.06×10^−5^	9.27×10^−6^	8%	1.48×10^−10^	1.4
	S432	T/**A**	3.51×10^−3^	1.84×10^−3^	7.15×10^−3^	4%	7.76×10^−5^	1.2
	S433	T/**A**	1.15×10^−3^	1.91×10^−3^	9.31×10^−4^	4%	5.23×10^−5^	1.2
	S1106	G/**A**	4.47×10^−8^	2.21×10^−7^	4.62×10^−6^	11%	7.11×10^−13^	1.6
	S1688	A/**T**	5.01×10^−8^	6.24×10^−7^	2.02×10^−6^	10%	3.84×10^−12^	1.8

a Only significant polymorphic sites are shown.

b Alleles in series are listed for each polymorphism, and favorable alleles (higher RAPA and RPC) are indicated in bold.

InDel170 allelic series: 1, no deletion; 2, 6 bp deletion; 3, 8 bp deletion; 4, 9 bp deletion; 5, 10 bp deletion. InDel423 allelic series: 1, 1 bp deletion; 2, 4 bp deletion; 0, 0 bp deletion.

c 2008NJ and 2008HN denote the 2008 field experiments at Nanjing and Henan, China. *P* values are from an association analysis performed using the mixed model, incorporating population structure and kinship, using data from two years and two sites.

d Phenotypic data across three environments were used for the analysis. *R^2^* values from multiple linear regression (MLR) of the data showing the percentage phenotypic variation explained.

e *P* value from the MLR analysis of the data across three environments.

f Fold change between two of the most differentiated haplotype classes.

ns: not significant at *P* = 0.05; RAPA: relative acid phosphatase activity in two P conditions; RPC: relative P concentration in two P conditions.

### Various *GmACP1* haplotypes display variation in gene expression and acid phosphatase activity

**Figure 6:**
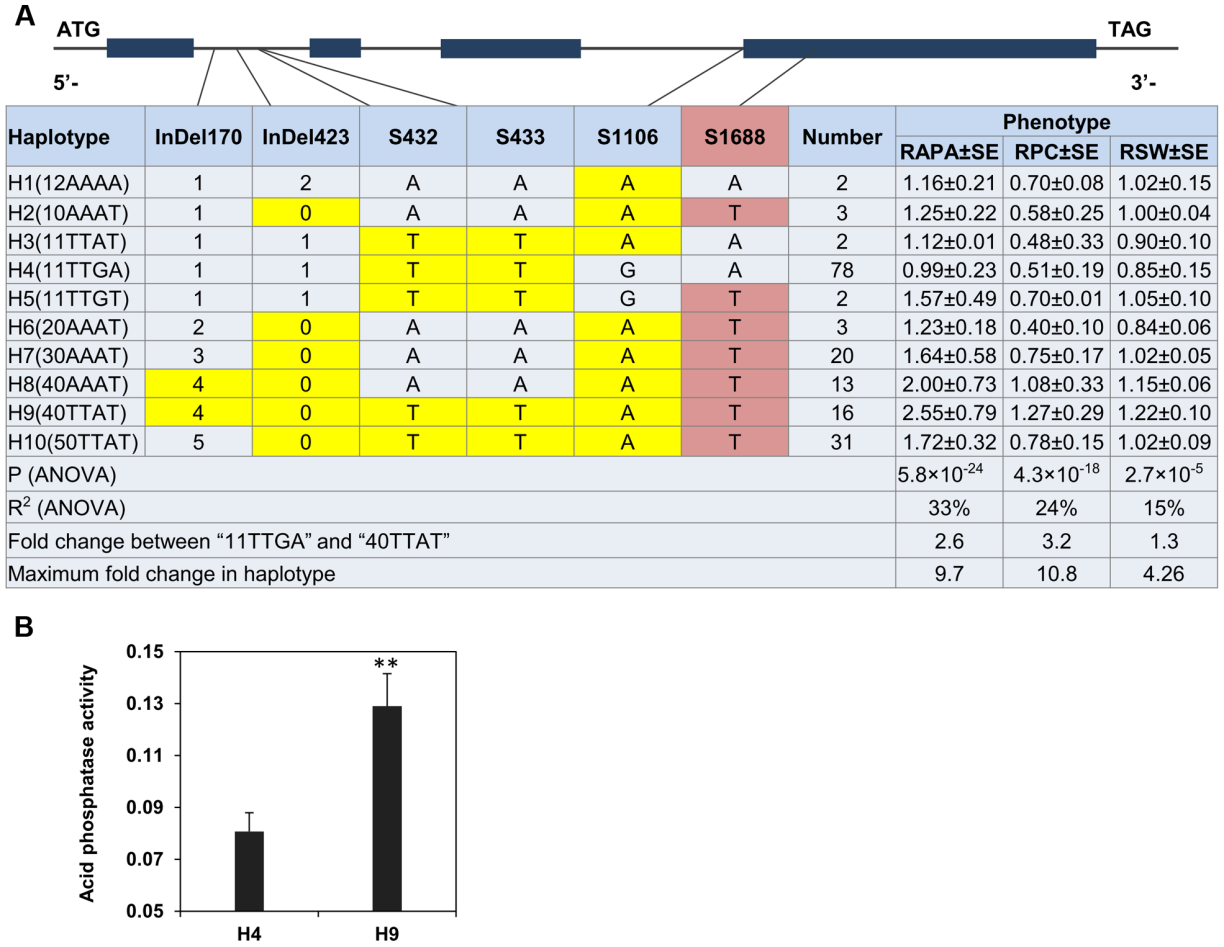
Haplotype analysis and acid phosphatase activity of proteins from two different haplotypes of *GmACP1* .(A) Haplotype analysis of the *GmACP1* gene region. The haplotype is shown as a linear combination of alleles (InDel170, InDel423, S432, S433, S1106 and S1688), and only the observed haplotypes are listed. The effects of these haplotypes were estimated based on the mean phenotypic values. The colored cells represent the favorable alleles, and the red-shaded cells represent polymorphisms that result in amino acid substitutions. Fold change between “11TTGA” and “40TTAT” represents the comparison between the best and the worst haplotypes as predicted by component allelic effects. RAPA: relative acid phosphatase activity under −P and +P; RPC: relative P concentration under −P and +P; RSW: relative 100-seed weight under −P and +P. (B) Acid phosphatase activity of proteins from different haplotypes of *GmACP1* expressed in *E. coli*. H4 denotes the haplotype “11TTGA” and H9 denotes the favorable haplotype “40TTAT”. ** indicates a significant difference at *P* = 0.01. The error bars represent the standard errors of three independent repetitions.

The strong LD of the six probable causative sites may cause overlapping effects. Therefore, the *GmACP1* haplotypes were used to determine the joint effects of the six putative causative sites related to P efficiency. Ten haplotype classes were observed in the natural population (H1–H10) (Figure 6A). The RAPA and RPC values were highest when the favorable alleles were combined into H9 (40TTAT). Overall, the ten haplotypes explained 33% of the phenotypic variation (*P* = 5.81×10^−43^, MLR) for the RAPA, and a 2.6-fold difference was observed between two of the most differentiated haplotype classes. Specifically, haplotype H9 (40TTAT) was observed in 16 soybean accessions, whereas H4 was observed in 78 soybean accessions. Notably, the proteins encoded by H9 and H4 differ by only one amino acid, phenylalanine vs. tyrosine. The acid phosphatase activity of the protein encoded by haplotype H9 expressed *in vitro* was 1.6-fold higher than the protein encoded by haplotype H4 (Figure 6B).

**Figure 7:**
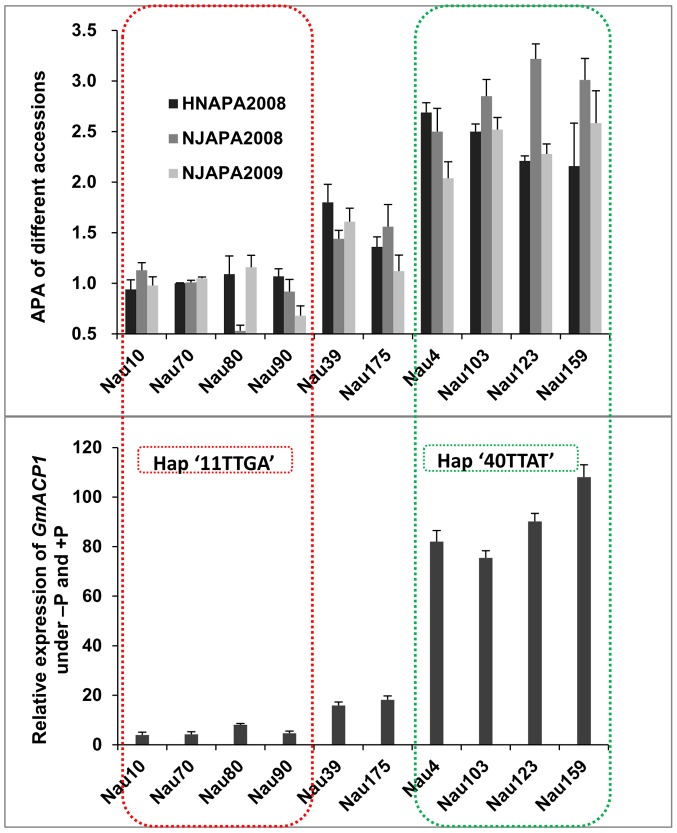
Relationships between the acid phosphatase activities (APA), *GmACP1* expression levels and haplotypes in the different accessions. The top panel corresponds to the APA from different accessions. The Y-axis denotes the relative APA (−P/+P). HNAPA2008 and NJAPA2008 denote the phenotypic data obtained in Henan and Nanjing in 2008. Nau10, Nau70, Nau80 and Nau90 are accessions with low P efficiency; Nau4, Nau103, Nau123 and Nau159 are accessions with high P efficiency; and Nau39 and Nau175 are accessions with moderate P efficiency. The bottom panel shows the *GmACP1* qRT-PCR data at seven days following low P stress. The Y-axis denotes the relative expression of *GmACP1* under −P and +P. The accessions marked by green boxes have the favorable haplotype “40TTAT” for P efficiency whereas the accessions marked by red boxes have the unfavorable haplotype.

Expression profiling of *GmACP1* in soybean roots after seven days of low-P stress indicated that *GmACP1* expression correlated well with the RAPA under various P conditions. Accessions with the favorable haplotype ‘40TTAT’, which have a 6–10 bp deletion in intron 1 and a nucleotide transition in exon 4, showed higher *GmACP1* expression levels (Figure 7), suggesting that the variation among the different haplotypes was responsible for the diverse APA in the soybean accessions. Based on the transformation of the soybean hairy roots with *GmACP1* and the strong association between *GmACP1* polymorphisms and traits related to soybean P efficiency, we speculated that *GmACP1* is a key P efficiency related gene in soybean. To our knowledge, this is the first P efficiency gene that has been cloned based on genetic mapping and genomic sequence data in soybean.

### Development of a functional marker for soybean P efficiency

**Figure 8:**
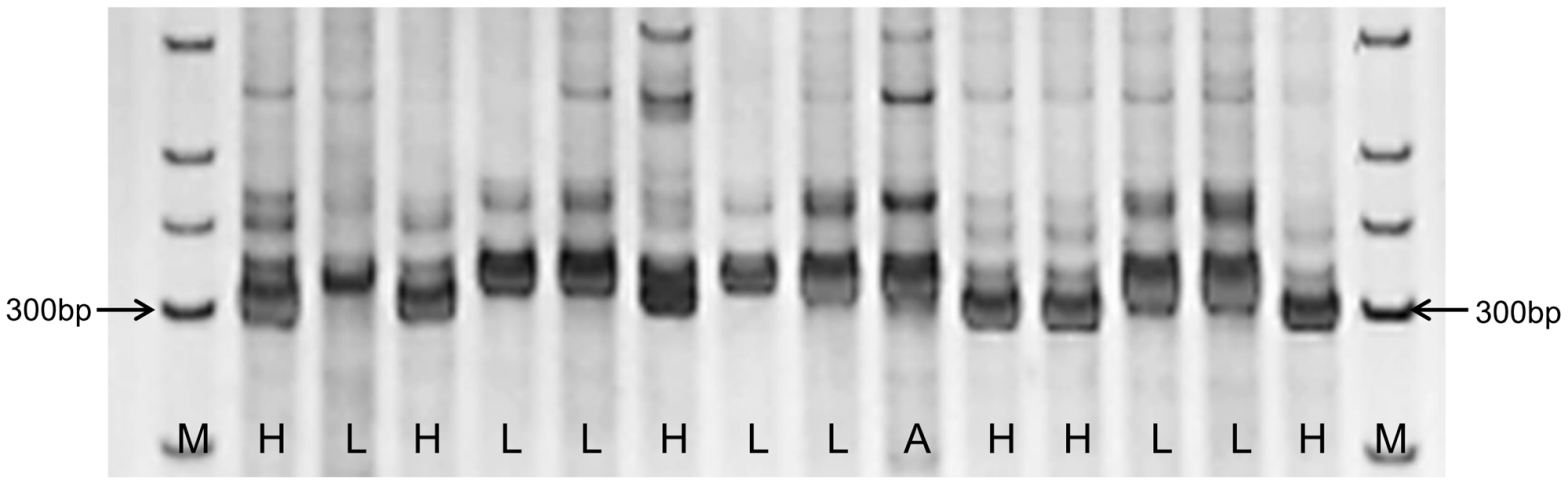
PCR assay of 14 soybean accessions using the InDel170 marker. The samples are shown in Table S2. L denotes the accessions with low P efficiency, H denotes the accessions with high P efficiency, and A represents the accession with moderate P efficiency. M, marker.

In this study, PCR markers for Indel170 were assayed in 14 soybean accessions, representing varieties with high and low P efficiencies, which were previously identified using conventional selection studies [12], [14], [46], [47] (Table S2). As shown in Figure 8, all seven accessions with low P efficiencies produced a 312 bp amplicon, as did one variety with moderate P efficiency that did not carry the deletion allele (Figure 8). The remaining six accessions with high P efficiencies produced amplicons of 302–306 bp because of the deletion of the Indel170 site. These results confirmed that Indel170 is highly associated with P efficiency and validated Indel170 as a functional marker.

## Discussion

Since P is an essential macro-element for all living cells, it is indispensable in entire ecosystem, especially in agricultural production systems [11]. Low soil P availability has a profound influence on global agriculture and food production [3]. Crops with high P efficiency need to be developed to maximize yield in low-input systems such as poor soils [48]. This development can be achieved by the selection of cultivars with enhanced phosphate-uptake or phosphate-use efficiency by traditional plant breeding programs or genetic engineering [49], [50].

Recently, it has been shown that the capacity of plants to scavenge P from soils could be increased by upregulating the proton-translocating pyrophosphatases (H^+^-PPases). For example, overexpression of H^+^-PPases in *Arabidopsis*, tomato, rice, alfalfa or cotton leads to more elaborate root systems and increased production of leaves, fruits and seeds [51]. Plants possess a large number of adaptive responses to phosphate limitation, which are avenues to enhance the phosphate scavenging abilities; therefore, more related genes should be characterized.

Although several P-efficiency-related QTLs have been mapped in *Arabidopsis* [52], rice [4], wheat [53], maize [50] and soybean [12], [14], [15], few of these QTLs have been cloned. Based on previous mapping of the soybean P efficiency QTL [14], [15], we combined linkage mapping, association mapping, bioinformatics and expression analysis to clone a P efficiency QTL, *qPE8*. Using *in vitro* expression and hairy root transformation we confirmed the function of the gene *GmACP1*. Finally, we identified the elite alleles and haplotypes of *GmACP1*, and developed valuable markers for breeding applications. This strategy could be useful for the dissection and cloning of the resting P efficiency QTL in the soybean genome and the P efficiency QTL in other plants.

### *GmACP1* functions as an acid phosphatase

The cloned soybean P efficiency gene *GmACP1*, showed significantly increased expression after P starvation (Figure 3 and 7). Comparison of the deduced GmACP1 amino acid sequence with related proteins (Figure S4) and a protein activity assay (Figure 4B) confirmed that *GmACP1* encodes an acid phosphatase.

Acid phosphatases are a family of enzymes that are widespread in plants, animals, algae and fungi [54]. They can catalyze the hydrolysis of various phosphomonesters in acidic medium to release an inorganic phosphate and play a pivotal role in P metabolism [55]. In the past decades, studies have revealed that acid phosphatase activity (APA) could be used as a diagnostic criterion for human diseases [54], [56], algae and plant P deficiency [57], [58]. In animals, acid phosphatases are normally found at low concentrations. However, pronounced changes in their synthesis occur in particular diseases, such as prostate cancer, bone dysplasia etc., where unusually high or low enzyme expression is seen as part of the pathophysiological process [56], [59]. Phosphatase activity has also been used as a P deficiency indicator in algae and in natural plankton populations [58]. In plant, for example, tomato (*Lycopersicon esculentum*) acid phosphatase gene *LePS2*, and the bean (*Phaseolus vulgaris*) acid phosphatase genes *PvPS2* were specifically induced upon P starvation [39], [40]. We observed also that P starvation increased the soybean root expression of *GmACP1* 110-fold relative to the control (Figure 3). Furthermore, in the 192 soybean accessions, the leaf APA was significantly associated with leaf P content (PC) (*P*<0.0001), suggesting that the leaf APA is also a diagnostic criterion for P deficiency in soybean and can be used to screen for high P efficiency germplasms in soybean.

In addition, acid phosphatases can effectively improve the acquisition and utilization of organic P complexes [55]. Therefore, the overexpression of genes encoding acid phosphatases is an attractive paradigm to develop plants with higher P efficiencies [2]. Wang *et al* (2009) overexpressed an *Arabidopsis* purple acid phosphatase gene (*AtPAP15*) in soybean hairy roots and found that the APA was increased by 1.5-fold in transgenic hairy roots [6]; they also overexpressed *AtPAP15* in soybean plants and observed significantly improved P efficiencies and yields. By overexpression in bean hairy roots and *Arabidopsis*, Liang *et al* (2012) observed that the putative bean protein phosphatase *PvS2:1* was involved in root growth and P accumulation [60]. In this study, the APA, root P content and root dry weight were significantly increased in the transgenic hairy roots overexpressing *GmACP1* (Figure 5), indicating that the overexpression of *GmACP1* could facilitate the uptake and use of organic phosphate in the culture solution by the transgenic soybean hairy roots.

Comprehensive analyses of transcripts in plants such as *Arabidopsis* [61] and white lupine (*Lupinus albus*) [62] have revealed the functional classification of the P-responsive genes in diverse metabolic pathways, including transcriptional regulation, ion transport, signal transduction, and other processes related to growth and development, indicating that the plant response to P deficiency is controlled by a complex network. The further characterization of the key genes in this network would provide deeper insight into the regulatory mechanisms involved in plant responses to low-P stress.

### Favorable haplotypes and alleles for high P efficiency

Stress resistance of an organism is a consequence of long-term co-evolution process. Resistance genes must maintain high mutation rates and high levels of nucleotide diversity to withstand environmental stresses [63]. SNPs, which include single base pair changes and small indels, are abundant and relatively stable in the genome, and they have been discovered within genes underlying observed traits [64]. However, SNPs within a narrow region are typically in high LD, i.e., adjacent SNP alleles on the same chromosome are strongly correlated [65]. Therefore, haplotype association is likely to be more powerful in the presence of LD, which could compensate for the bi-allelic limitation of SNPs and substantially improve the efficiency of QTL mapping [66], [67]. Sequences and haplotype analysis of a candidate gene for aluminum (Al) accumulation, *Nrat1*, in 383 diverse rice accessions identified a tolerant haplotype that explained 40% of the Al tolerance variation and three non-synonymous mutations in *Nrat1* that were predictive of Al sensitivity [28]. Association of the natural protein haplotypes of Heavy Metal ATPase 3 (HMA3) from 149 *Arabidopsis* accessions with leaf cadmium (Cd) accumulation and genetic complementation experiments identified five active haplotypes [68]. The elevated leaf Cd accumulation was associated with the reduced function of HMA3 caused by a nonsense mutation and polymorphisms that changed two specific amino acids [68]. Other recent studies include the identification of the downy mildew resistance genes in *Arabidopsis* [69] and the cyst nematode resistance gene in soybean [70]. Although some P efficiency genes have been characterized [11], to our knowledge, the natural variation and haplotype analysis of these genes and the possible underlying mechanism have not been reported.

In this study, haplotype analysis of *GmACP1* gene revealed ten haplotypes (H1 to H10) (Figure 6A). The RAPA, RPC and RSW of these ten haplotypes were significantly associated with each other, indicating that the P efficiency gene could contribute to seed yield and has therefore been retained during soybean domestication and breeding. Notably, the *in vitro* expressed protein encoded by the favorable haplotype H9 had 1.6-fold higher enzymatic activity than the protein encoded by the unfavorable haplotype H4 (Figure 6B), indicating that haplotype H9 might be significant for engineering plants with higher P efficiencies. Further studies on the origins of this favorable haplotype would be interesting.

In this study, the 192 soybean accessions used to identify the favorable haplotype of *GmACP1* were selected to represent all six ecological regions of soybean cultivation in China and soybeans with differences in P efficiency. However, as more than 20,000 soybean accessions are preserved [71], other elite alleles of *GmACP1* might be discovered using the stored soybean germplasms. These results can be incorporated into breeding efforts to design plants for more sustainable agriculture and a healthier environment.

## Materials and Methods

### Plant materials and field experiments

To map QTLs for P efficiency, a segregating soybean population consisting of 152 F_8∶10_ recombinant inbred lines (RILs) were used. These RILs were derived from a cross between the ‘Nannong 94–156’ variety that possessed high P efficiency and the ‘Bogao’ variety with low P efficiency. For the association mapping experiments, a natural population of 192 soybean accessions (Table S3), including landraces, cultivars and breeding lines collected from latitudes 53 to 24°N and longitudes 134 to 97°E in China, was used. These accessions were selected because they represented all six ecological regions of soybean cultivation in China and soybeans with differences in P efficiency. The seeds of each accession were obtained from the Germplasm Storage in the National Center for Soybean Improvement (Nanjing, China).

For the linkage mapping analyses, greenhouse trials using 152 RILs to map QTLs related to P efficiency were performed over two years at the Jiangpu Station in Nanjing, China, in 2006 and 2007 (all field experiments were conducted from June to October). For the association mapping studies, two greenhouse trials using the 192 accessions were performed at the Jiangpu Station in Nanjing, China and the Maozhuang Station in Henan, China in 2008, and a repeat experiment was performed in Nanjing in 2009. These experiments used a completely randomized design with a split-plot restriction. The primary plots included treatments (low P and normal P), and the subplots were the soybean genotypes. Three replicates were performed, with six plants per replicate. The soil had a very low P concentration of 2.51 mg kg^−1^ available P and contained 0.2 g kg^−1^ total nitrogen, 52.4 mg kg^−1^ available K and 12.8 g kg^−1^ organic matter [9]. To evaluate the plant responses to low P availability, 60 mg kg^−1^ H_2_NCONH_2_ and 20 mg kg^−1^ KCl or KH_2_PO_4_ were applied to the low P and high P treatment pots in different growth stages, respectively.

### Measurement of acid phosphatase activity, P concentration and plant dry weight

Leaf samples weighing approximately 0.1 g taken from soybean plants in the field experiments or soybean hairy root samples weighing 0.1 g were ground in liquid nitrogen and macerated in 1 mL of extraction buffer (50 mM sodium acetate buffer, pH 5.0). The extract was then centrifuged at 16,000×g for 10 min at 4°C. The supernatant (10 µL) was mixed with 490 µL of extraction buffer and used to assay the acid phosphatase activity (APA) with *ρ*-nitrophenol phosphate (*ρ*-NPP) as the substrate [39]. The acid phosphatase activity was measured at 37°C for 10 min, and the reaction was terminated using 1 mL of 2 M NaOH. The OD at 410 nm was measured in a spectrophotometer. *In situ* staining for APA was performed by culturing whole transgenic roots in B&D solution [72] with *ρ*-NPP for one week at 27°C.

The plant enzymes were deactivated by heat-induced denaturation at 105°C for 60 min; the samples were then oven-dried at 65°C for three days. The dried samples were milled and subsequently digested with concentrated H_2_SO_4_ and H_2_O_2_ to facilitate the determination of P concentration using the molybdate-blue colorimetric method [73]. The P efficiency in the hairy root (root P-use efficiency) was defined as the mass (mg) of root dry weight produced per mg of P absorbed by roots [74].

### QTL mapping and analysis of gene synteny

For the linkage mapping analysis, the composite interval mapping (CIM) program of WinQTLCart version 2.5 [75] was used to detect QTLs for traits related to P efficiency using the 152 RILs. For each trait, empirical thresholds were computed using the permutation test (1,000 permutations, overall error level 5%) for CIM [76]. The confidence intervals were set as the map interval that corresponded to a 1-LOD decline on either side of the LOD peak. A genetic map comprising 306 markers was constructed and used for linkage mapping [14]. The genes in the target genomic region were predicted using the soybean genome information (http://soybase.org/SequenceIntro.php). The candidate genes related to P efficiency were identified by querying the predicted genes using BLASTP of the protein database (http://www.ebi.ac.uk/Tools/sss/ncbiblast/), and synteny comparisons were performed between soybean and other dicotyledonous plants.

### Determination of transcript levels by qRT-PCR

The candidate gene expression was analyzed using hydroponically grown plants. The seeds were surface-sterilized with chlorine and germinated in sterile vermiculite. When the cotyledons were fully expanded, the soybean seedlings were selected and transferred to modified one-half Hoagland's nutrient solution supplemented with 500 µM KH_2_PO_4_ for seven days. The seedlings were then transferred to modified one-half Hoagland's nutrient solution lacking P for seven days. Treatment with 500 µM KH_2_PO_4_ was used as a control. The roots and shoots were sampled and stored at −70°C for further use. The total RNA was isolated from the roots of soybean plants using the RNA simple Total RNA Kit (DP419, TIANGEN, Beijing, China) and treated with 10 units of RNase-free DNase I (TaKaRa, Japan). The first strand of cDNA was synthesized using the SuperScript III First Strand Synthesis System (Invitrogen, USA). Gene expression was determined by qRT-PCR assays using the ABI 7500 system (Applied Biosystems, Foster City, USA). The PCR reactions contained 50 ng of the first cDNA strand, 0.5 µL of 10 µmol L^−1^ gene-specific primers (Table S1), and 10 µL of the real-time PCR SYBR MIX (QPK-201; TOYOBO). The PCR conditions were as follows: 95°C for 5 min and 40 cycles at 95°C for 15 s and 60°C for 60 s. The soybean *tubulin* gene (GenBank: AY907703.1) was amplified as a positive control, and a negative control reaction was performed using water instead of the cDNA. Three replicates were performed for each reaction, and the data were analyzed using the ABI 7500 Sequence Detection System (SDS) software version 1.4.0. The normalized expression, reported as fold changes, was calculated for each sample as ^ΔΔ^CT = (C_T, Target_−C_T, Tubulin_) _genotype_−(C_T, Target_−C_T, Tubulin_) _calibrator_ [77].

### DNA sequencing and genotyping

The genomic DNA was isolated from the bulked leaf tissues of eight to 10 plants, as previously described [78]. The PCR primers for the different germplasms were designed using the Primer 3 online tool (http://frodo.wi.mit.edu/primer3/). To identify SNPs in the *GmACP1* gene, we sequenced approximately 3,300 bp, including part of the promoter, the 5′-UTR, the complete CDS, three introns and part of the 3′-UTR in ten representative P-efficient accessions that displayed differences in P efficiency. These regions were amplified by PCR using the following primer pairs: (1) 5′-AGCATCCACAGAAAAATCCC/AGAACGAGGGAATAAAAGGG-3′ at 58°C (annealing temperature), (2) 5′-TTATTCCCTCGTTCTGCTCT/GAATAAGGCTCTGTTTGGGT-3′ at 58°C, (3) 5′-TCACTTTTAGCAGCACTCTC/CTGGTTGAACAAATCGGTGA-3′ at 60°C, (4) 5′-TCACTTTTAGCAGCACTCTC/CTGGTTGAACAAATCGGTGA-3′ at 58°C, and (5) 5′-ATGAAAAGAGATGGATGCTA/AATACAAGTCCAATAACCTA-3′ at 58°C. The PCR reactions were conducted in 50 µL volumes using the *Ex*Taq polymerase (TAKARA, Kobe, Japan), following the manufacturer's recommendations. The PCRs were performed using a PTC-225 thermal cycler (MJ Research, Watertown, MA) as follows: 1 cycle of 5 min at 94°C; 30 cycles of 40 s at 94°C, 1 min at the appropriate annealing temperature of the specific primer pair and 1 min at 72°C; and 1 cycle of 10 min at 72°C. The DNA sequencing was performed at Takara (Dalian, China), and each PCR fragment was sequenced in both directions. All sequences were verified manually, and all observed singletons were verified by sequencing the newly amplified fragments. The sequences were aligned using the ClustalX software version 1.83 [79]. The polymorphism data were analyzed using the DnaSP software version 4.10 [80] to identify sequence variation. The identified SNPs with minor allele frequencies of at least 5% were genotyped in a larger sample from the population. The LD analysis was performed using the HapView 4.0 software program [81] on the entire *GmACP1* sequence; a window size of 50 bp was used to plot the average *D′* against the distance (base pairs). The significance of LD between the sites was determined using Fisher's exact test.

### Association analysis

The 192 soybean accessions were genotyped with 82 unlinked SSRs that provided representative coverage of the soybean genome. The employed SSR markers are publicly available (http://bldg6.arsusda.gov/cregan/soy_map1.html). The population structure was inferred from the SSR data using the STRUCTURE software, version 2.2 [82]. Six independent runs were implemented using the following parameters: number of populations (*K*) from 1 to 10, burn-in time and Markov-chain Monte Carlo replication numbers set to 500,000, model of admixture and correlated allele frequencies. The K value was determined by LnP (D) in the STRUCTURE output, and the population structure at *K* = 2 was used for the association analysis, as previously reported [83]. STRUCTURE produces a *Q* matrix that lists the estimated membership coefficients for each individual in a cluster; this information was used in the subsequent association analysis. The relative kinship matrix was calculated using 1,536 SNP markers [35] for the 192 accessions by SPAGeDi, which is a program that analyzes the spatial genetic structure at the individual or population levels [84]. The negative kinship values between individuals were set to zero.

Both the genome-wide association and candidate-gene association analyses were performed based on the 192 natural soybean accessions and the 1,536 SNPs [35] using a mixed-model approach that controlled for complex population structure and pedigree relationships [85], implemented in TASSEL version 4 [45]. This approach simultaneously accounted for multiple levels of relatedness based on random genetic markers, which were used to establish the population structure and kinship matrix. All polymorphisms (frequency >5%) were tested, and the *P*-value for each site was estimated based on 1,000 permutations of the dataset under a mixed linear model (MLM). Markers were defined as being significantly associated with traits on the basis of a significant association threshold (−Log*P*≥2.00, *P*≤0.01). Associations for the relative acid phosphatase activity (RAPA) and relative P concentration (RPC) were performed on the 192 soybean accessions; only the significant association sites were reported. We performed a multiple linear regression (MLR) using SAS version 9.0 (GLM) (SAS Institute Inc., Cary, NC, USA) to detect the effect of *GmACP1* polymorphisms and haplotypes in this natural soybean population. Furthermore, we used a 5-fold cross validation to estimate accuracy of the algorithm (MLR). We divided the phenotypic data into five segments, four of which were used for training, whereas one segment was omitted and used for testing. This was performed 1000 times; in the first instance, the first segment was used for testing and the remainder was used for training; then, the second segment was used for testing and the remaining segments were used for training, and so on.

For the regional association mapping studies, the 192 soybean accessions were genotyped with 18 SSR markers and 22 SNP markers based on the soybean physical map (http://soybase.org/gbrowse/cgi-bin/gbrowse/gmax1.01/#search). The regional association analysis was performed as described above.

### Expression and purification of GmACP1 in *E. coli*

The *GmACP1* coding sequences (including the sequences of high/low P efficiency haplotypes) were was cloned into the *pET28a* expression vector and expressed in *E. coli* (BL21) cells, as described by Baldwin *et al.* [39]. The recombinant clones contained His tags at both the N- and C-terminal ends of the GmACP1 peptide. The *E. coli* cells were induced with IPTG to produce the recombinant protein and then lysed by sonication and run through a Ni-affinity column, according to the manufacturer's recommendations (GenScript, TM0217). The purity of the affinity-purified GmACP1 protein was confirmed on an SDS-PAGE gel. The GmACP1 protein concentration was measured using the Bradford Protein Assay Kit (Bio-Rad, Hercules, CA). The reactions contained 150 ng of GmACP1 protein and 1 µg of synthetic substrate *ρ*-nitrophenyl phosphate in 500 µL of NaAc-HAc solution (100 mM). Control assays were performed using an extract from *E. coli* containing only pET-28a. All reactions were performed in triplicate. The GmACP1 phosphatase activity was measured at 37°C for 10 min with pH values ranging from 3.0 to 9.0.

### Soybean hairy root transformation

The *GmACP1* overexpression vector was constructed using the Gateway technology with the Clonase II Kit (Invitrogen, Carlsbad, CA). The *GmACP1* open reading frame (ORF) was amplified from the cDNA of soybean accession Kefeng No. 1, which is a variety with high P efficiency, using gene-specific primers (forward: 5′-GGGGACAAGTTTGTACAAAAAAGCAGGCTTCCAACATGTCTGGAACCGTGAT-3′, reverse: 5′-GGGGACCACTTTGTACAAGAAAGCTGGGTCTGGTCTA CTGGGAGGACT-3′). Two recombination reactions (BP and LR reactions) constitute the basis of the Gateway cloning technology. Finally, the amplified fragment was introduced into the pMDC83 vector according to the manufacturer's instructions and confirmed by sequencing. An empty vector was constructed using a single enzyme digestion method. The plasmid pMDC83 was digested with the restriction endonuclease *Kpn*I and re-annealed following the removal of the fragment of the recombination site between attR1 and attR2. This process was confirmed by PCR (forward: 5′-GGTTGGCCATGGAACAGGTA-3′, reverse: 5′-GAGGACCTCGA CTCTAGAAC-3′) and sequencing analyses. Next, the expression vector containing the *GmACP1* gene and empty vector were introduced into *Agrobacterium rhizogenes* K599 (kindly provided by Prof. Peter Gresshoff) through the freeze-thaw method. Soybean hairy root transformation was performed using the accession Kefeng No. 1, as previously described by Kereszt *et al.* [43]. The seeds were surface-sterilized with chlorine gas for 4 h prior to germination in vermiculite.

## Supporting Information

Figure S1 Phosphorus (P) efficiency related QTL mapped on chromosome 8 using 152 RILs in 2006 and 2007. The black arrow indicates the P efficiency related QTL (*qPE8*) mapped to the location of *GmACP1* on chromosome 8.
(TIF)

Figure S2 Quantile-quantile plots of estimated −log10 (*p*) for phosphorus efficiency related traits from association analysis based on MLM with *Q* and *K*. HNAPA2008 and NJAPA2008 denote the phenotypic data obtained in Henan and Nanjing in 2008. The black triangles represent the *P* values expected under the null distribution.
(TIF)

Figure S3 qRT-PCR of five candidate genes in two representative accessions with different phosphorus (P) efficiency values (the Y-axis denotes the gene expression levels). Nau3 (L) is an accession with low P efficiency, and Nau4 (H) is an accession with high P efficiency (gene annotation: *Glyma08g20700*, Calcineurin B; *Glyma08g20710*, Phospholipase D; *Glyma08g20800*, Putative Phosphatase; *Glyma08g20820*, Putative Phosphatase; and *Glyma08g20830*, Protein Phosphatase).
(TIF)

Figure S4 Comparison of GmACP1 with other related proteins. Invariant residues are shown in bold, and other conserved residues are highlighted. Alignment of the amino acid sequences revealed two peptide motifs that are conserved in the active site of the HAD and DDDD superfamilies of hydrolytic phosphotransferases. The Asp residue predicted to be transiently phosphorylated during the phosphate transfer reaction is indicated with an arrow. The abbreviations and GenBank accession numbers for the acid phosphatase sequences analyzed are as follows: *Phaseolus vulgaris* putative phosphatase (ABP52095); *Ricinus communis* putative phosphatase (XP_002527425); *Arabidopsis thaliana* putative phosphatase (NP 173213); *LePS2*, *Lycopersicon esculentum* (AAG40473); and *Zea mays* putative phosphatase (NP_001151156).
(TIF)

Figure S5 Linkage disequilibrium (LD) across *GmACP1* in 192 soybean accessions. The bp positions of the polymorphisms in the alignment are shown on the left. Lower left triangle: *P*-value derived from Fisher's exact test. Upper right triangle: D′ values.
(TIF)

Figure S6 Relationship between the acid phosphatase activity and the *GmACP1* polymorphisms/haplotype in the two parents. The parent with low P efficiency (Bogao) had the haplotype “11TTGA” and the parent with high P efficiency (Nannong94–156) had the favorable haplotype “40TTAT.” ** indicates significance at *P* = 0.01.
(TIF)

Figure S7 R^2^ of the relative acid phosphatase activity (RAPA) and relative phosphorus concentration (RPC) across three environments based on a 5-fold cross validation. The red arrows indicate the means calculated by multiple linear regression (MLR) analysis.
(TIF)

Table S1 Twenty-eight annotated genes between Sat_233 and BARC-039899-07603, five of which were considered putative candidate genes related to phosphorus efficiency (bold).
(DOCX)

Table S2 Fourteen previously reported soybean accessions with diverse phosphorus efficiencies.
(DOCX)

Table S3 Summary of the 192 soybean accessions used.
(DOCX)
