# Supplementary figures and images for: The Acid Phosphatase-Encoding Gene GmACP1 Contributes to Soybean Tolerance to Low-Phosphorus Stress
Source: PLoS Genet. 2014 Jan 2;10(1):e1004061. doi: 10.1371/journal.pgen.1004061 (PMC3879153; doi:10.1371/journal.pgen.1004061)

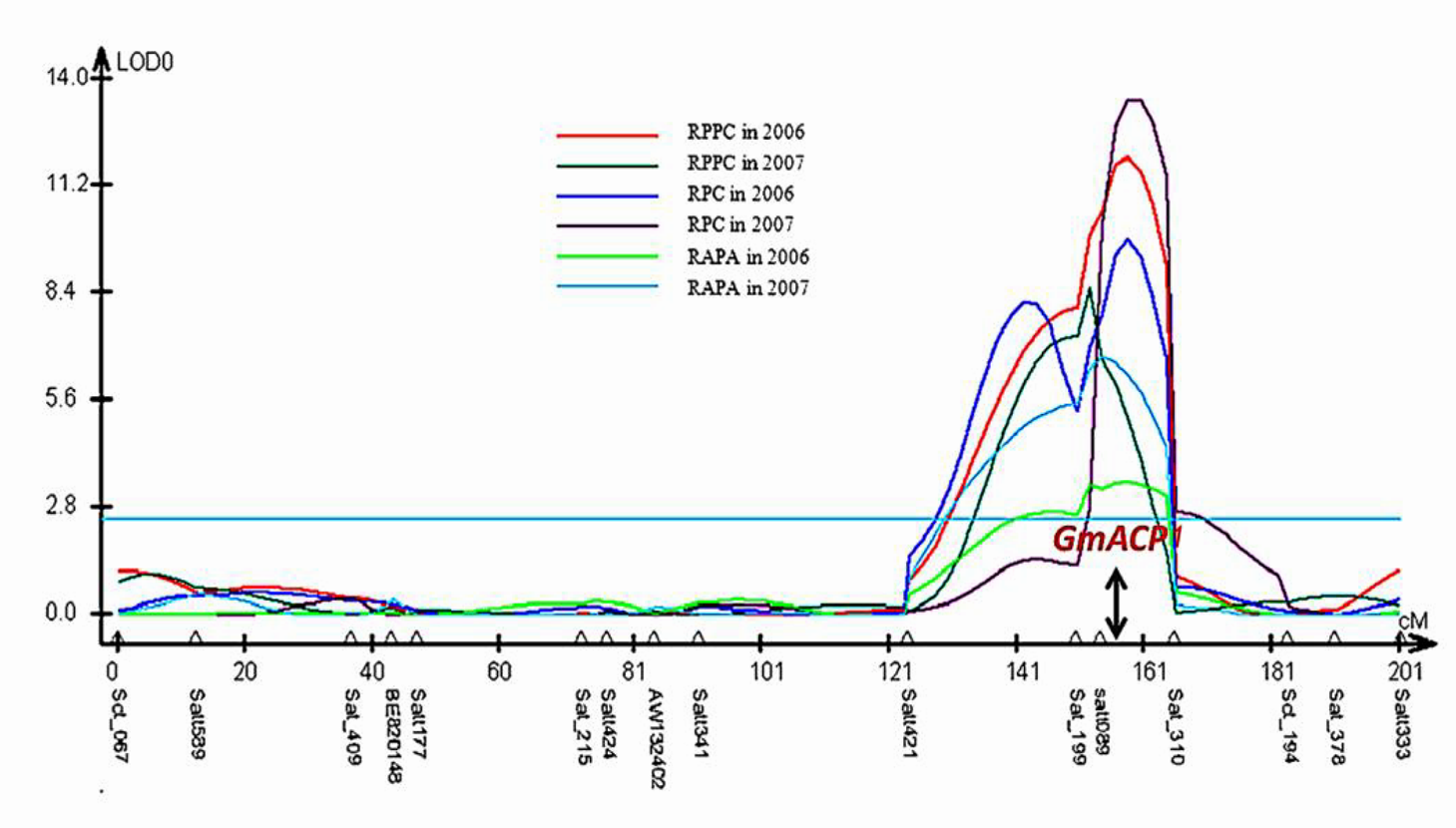

Supplement: Figure S1 — Phosphorus (P) efficiency related QTL mapped on chromosome 8 using 152 RILs in 2006 and 2007. The black arrow indicates the P efficiency related QTL (qPE8) mapped to the location of GmACP1 on chromosome 8. (TIF) [file pgen.1004061.s001.tif]

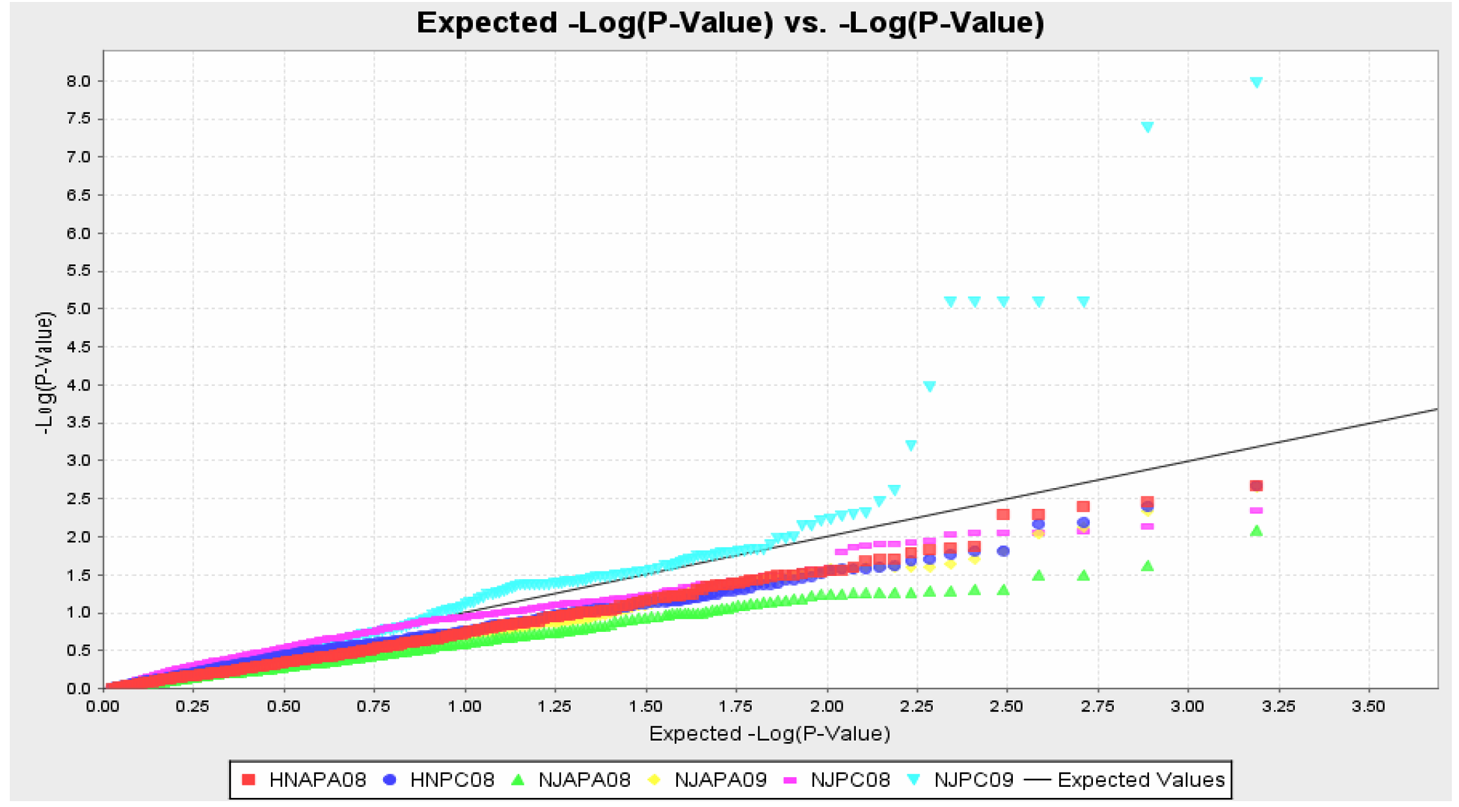

Supplement: Figure S2 — Quantile-quantile plots of estimated −log10 (p) for phosphorus efficiency related traits from association analysis based on MLM with Q and K. HNAPA2008 and NJAPA2008 denote the phenotypic data obtained in Henan and Nanjing in 2008. The black triangles represent the P values expected under the null distribution. (TIF) [file pgen.1004061.s002.tif]

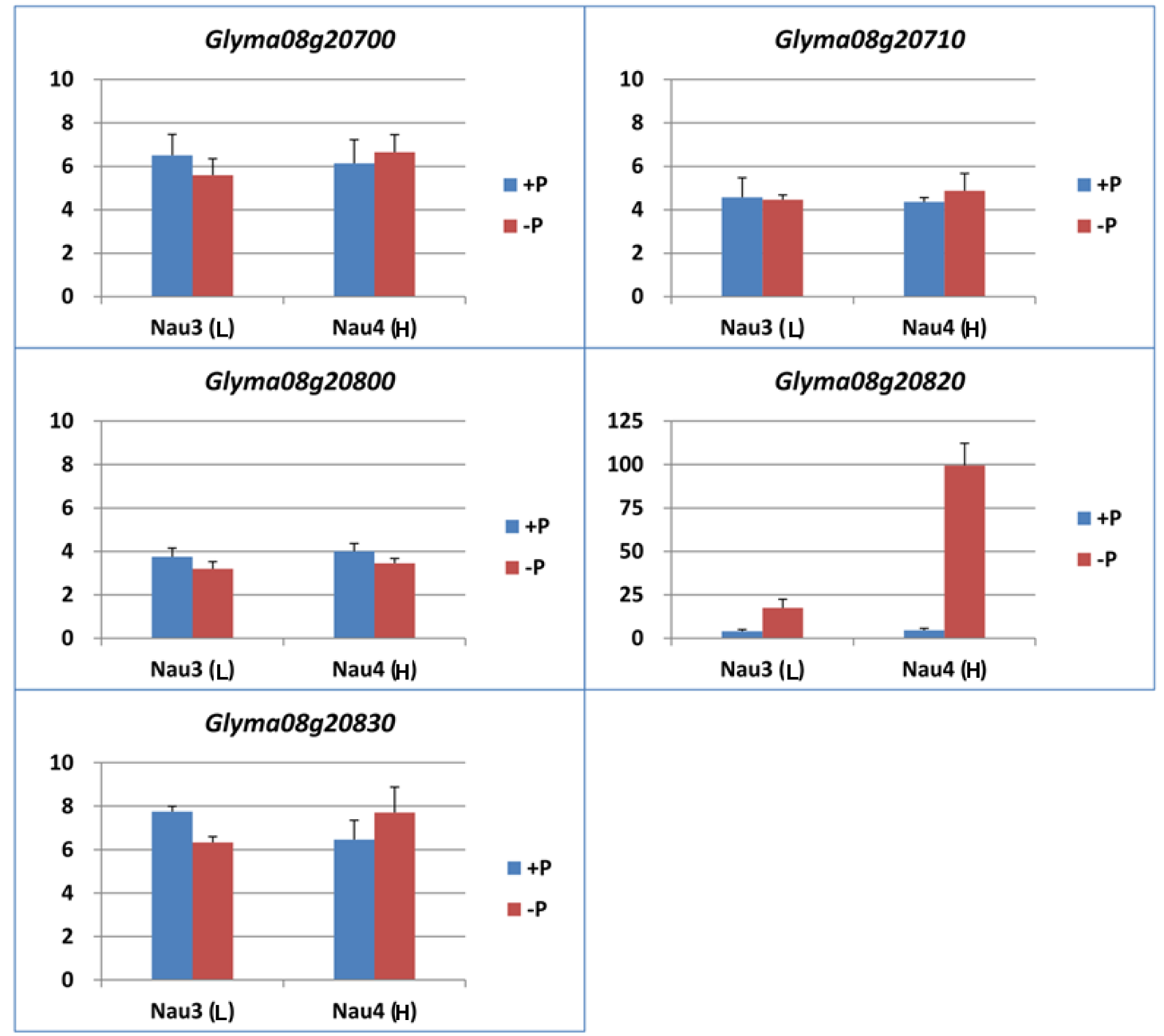

Supplement: Figure S3 — qRT-PCR of five candidate genes in two representative accessions with different phosphorus (P) efficiency values (the Y-axis denotes the gene expression levels). Nau3 (L) is an accession with low P efficiency, and Nau4 (H) is an accession with high P efficiency (gene annotation: Glyma08g20700, Calcineurin B; Glyma08g20710, Phospholipase D; Glyma08g20800, Putative Phosphatase; Glyma08g20820, Putative Phosphatase; and Glyma08g20830, Protein Phosphatase). (TIF) [file pgen.1004061.s003.tif]

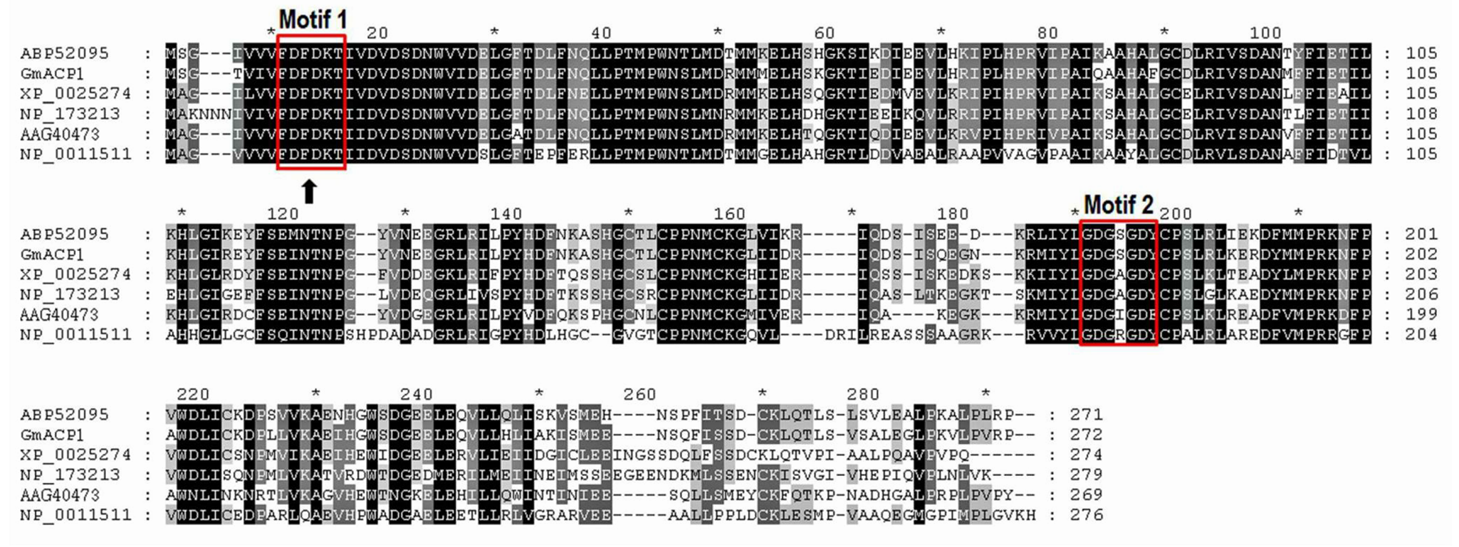

Supplement: Figure S4 — Comparison of GmACP1 with other related proteins. Invariant residues are shown in bold, and other conserved residues are highlighted. Alignment of the amino acid sequences revealed two peptide motifs that are conserved in the active site of the HAD and DDDD superfamilies of hydrolytic phosphotransferases. The Asp residue predicted to be transiently phosphorylated during the phosphate transfer reaction is indicated with an arrow. The abbreviations and GenBank accession numbers for the acid phosphatase sequences analyzed are as follows: Phaseolus vulgaris putative phosphatase (ABP52095); Ricinus communis putative phosphatase (XP_002527425); Arabidopsis thaliana putative phosphatase (NP 173213); LePS2, Lycopersicon esculentum (AAG40473); and Zea mays putative phosphatase (NP_001151156). (TIF) [file pgen.1004061.s004.tif]

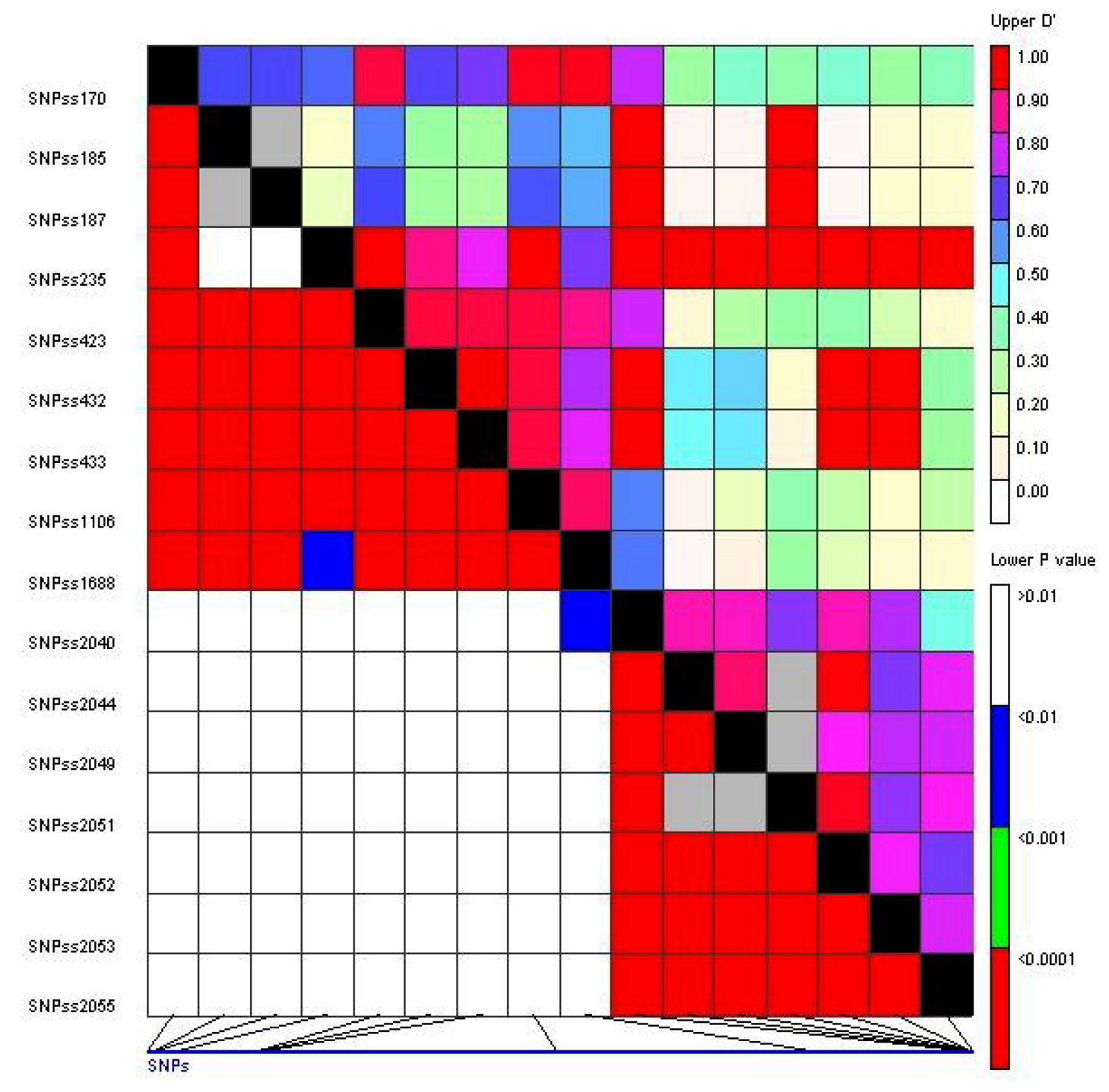

Supplement: Figure S5 — Linkage disequilibrium (LD) across GmACP1 in 192 soybean accessions. The bp positions of the polymorphisms in the alignment are shown on the left. Lower left triangle: P-value derived from Fisher's exact test. Upper right triangle: D′ values. (TIF) [file pgen.1004061.s005.tif]

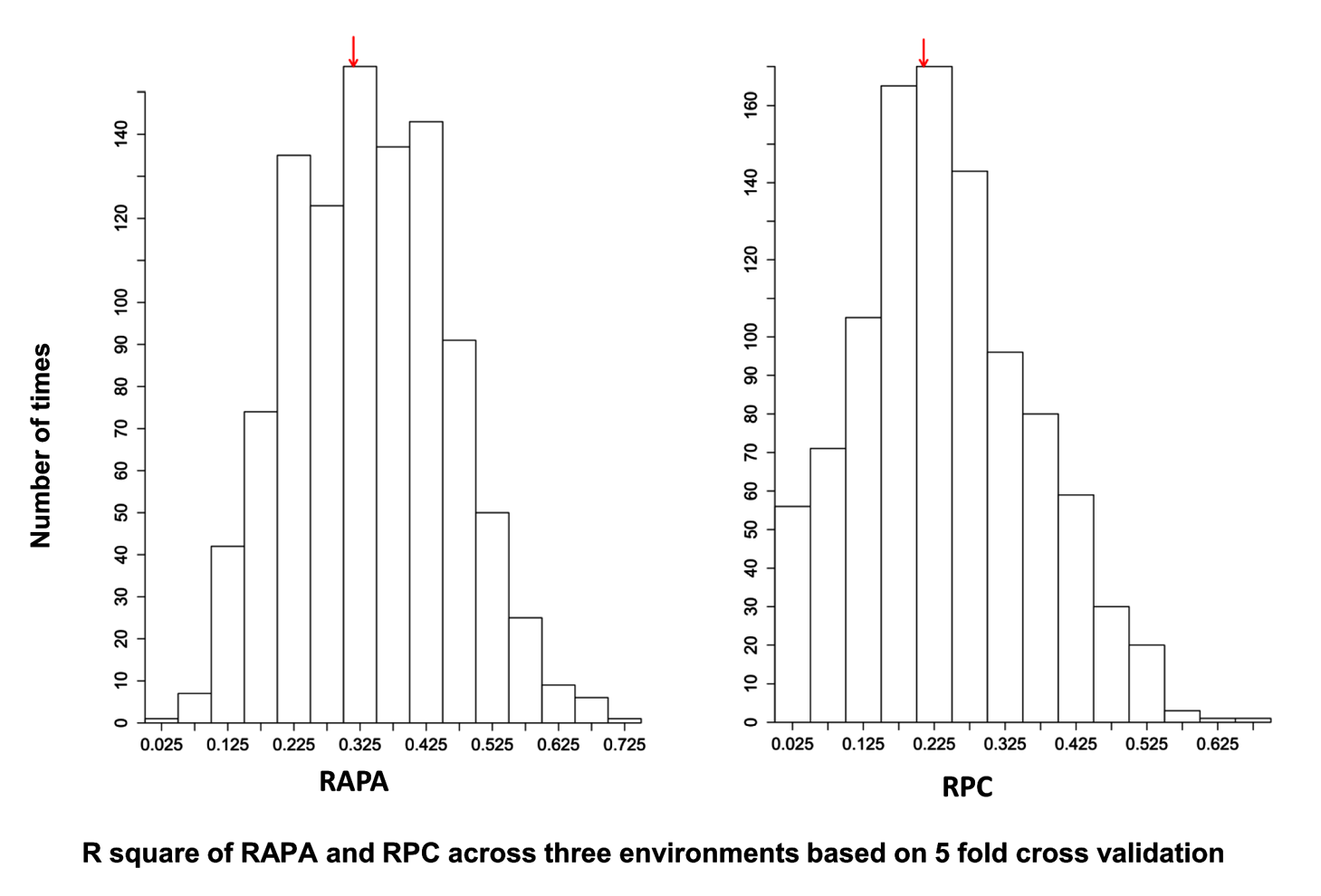

Supplement: Figure S7 — R2 of the relative acid phosphatase activity (RAPA) and relative phosphorus concentration (RPC) across three environments based on a 5-fold cross validation. The red arrows indicate the means calculated by multiple linear regression (MLR) analysis. (TIF) [file pgen.1004061.s007.tif]
