# Supplementary material for: The Acid Phosphatase-Encoding Gene GmACP1 Contributes to Soybean Tolerance to Low-Phosphorus Stress
Source: PLoS Genet. 2014 Jan 2;10(1):e1004061. doi: 10.1371/journal.pgen.1004061 (PMC3879153; doi:10.1371/journal.pgen.1004061)
Supplement: Table S1 — Twenty-eight annotated genes between Sat_233 and BARC-039899-07603, five of which were considered putative candidate genes related to phosphorus efficiency (bold). (DOCX) [file pgen.1004061.s008.docx]

Table S1. Twenty-eight annotated genes in Sat_233 and BARC-039899-07603, five of which were considered to represent putative candidate genes related to phosphorus efficiency (indicated in boldface).

| **Number** | **Name** | **Annotations** | **Primer sequences for real-time PCR (F/R)** |
| --- | --- | --- | --- |
| 1 | *Glyma08g20560* | Mitochondrial chaperonin |  |
| 2 | *Glyma08g20570* | Uncharacterized protein |  |
| 3 | *Glyma08g20580* | Leucine-rich repeat |  |
| 4 | *Glyma08g20590* | Protein tyrosine kinase | TACTCCACCCAGCTCCCATT/  CAACGTGCCCAGAAAAAAACA |
| 5 | *Glyma08g20600* | Glucose methanol choline oxidoreductase |  |
| 6 | *Glyma08g20610* | GTP-binding protein |  |
| 7 | *Glyma08g20620* | Protein kinase family protein |  |
| 8 | *Glyma08g20630* | Uncharacterized protein |  |
| 9 | *Glyma08g20640* | Endomembrane protein |  |
| 10 | *Glyma08g20650* | Beta-galactosidase |  |
| 11 | *Glyma08g20660* | uncharacterized protein |  |
| 12 | *Glyma08g20670* | ATP-dependent RNA helicase |  |
| 13 | *Glyma08g20680* | Uncharacterized protein |  |
| 14 | *Glyma08g20690* | Cytochrome P450 |  |
| 15 | ***Glyma08g20700*** | **Calcineurin B** | GCAGTGTTCATTCCGTTCATAGG/ CGGCGAGGGTGAGAATGT |
| 16 | ***Glyma08g20710*** | **Phospholipase D** | GCCATTGAGGGTGCGAAGTA/  TCCCAAAGCATAACCCTCACA |
| 17 | *Glyma08g20720* | Prenylated rab acceptor 1 family protein |  |
| 18 | *Glyma08g20730* | RNA binding protein |  |
| 19 | *Glyma08g20740* | Alpha-helical protein |  |
| 20 | *Glyma08g20750* | Serine/threonine protein kinase | GGGTGGTTGTGTTGGATGCT/  CGGTTTCCTCACTACGTTACTTTG |
| 21 | *Glyma08g20760* | Uncharacterized protein |  |
| 22 | *Glyma08g20770* | ABC transporter |  |
| 23 | *Glyma08g20780* | ABC transporter |  |
| 24 | *Glyma08g20790* | Uncharacterized protein |  |
| 25 | ***Glyma08g20800*** | **Putative phosphatase** | AAGGGTGCAAGCAGAAGCA/ GCGAGCCGCACAAGTCTTA |
| 26 | *Glyma08g20810* | Haloacid dehalogenase |  |
| 27 | ***Glyma08g20820*** | **Putative phosphatase** | GAGAGAGACTATATGATGCCAAGGAA/ AACTGGGAGGACTTTAGGCAAAC |
| 28 | ***Glyma08g20830*** | **Protein phosphatase** | GCGGCAGTCAGCATTATGTG/ TCTTCCATCCTTCCCAAATTTTAT |
