# Supplementary material for: The Acid Phosphatase-Encoding Gene GmACP1 Contributes to Soybean Tolerance to Low-Phosphorus Stress
Source: PLoS Genet. 2014 Jan 2;10(1):e1004061. doi: 10.1371/journal.pgen.1004061 (PMC3879153; doi:10.1371/journal.pgen.1004061)
Supplement: Table S2 — Fourteen previously reported soybean accessions with diverse phosphorus efficiencies. (DOCX) [file pgen.1004061.s009.docx]

Table S2. Fourteen previously reported soybean accessions with diverse phosphorus (P) efficiency.

| **No.** | **Accession** | **Reaction to P** | **Reference** |
| --- | --- | --- | --- |
| 1 | 1138-2 | High P efficiency | Li et al., 2005 |
| 2 | Ju rong da bian dou | Low P efficiency | Liu et al., 2005 |
| 3 | Qi huang 1 | High P efficiency | Liu et al., 2005 |
| 4 | Tie feng 3 | Low P efficiency | Ao et al., 2008 |
| 5 | Ning hai wan huang dou | Low P efficiency | Liu et al., 2005 |
| 6 | Xin chang liu yue dou | High P efficiency | Liu et al., 2005 |
| 7 | Su xie 1 | Low P efficiency | Liu et al., 2005 |
| 8 | Bo gao | Low P efficiency | Zhang et al., 2009 |
| 9 | Wen shang gun long zhu | Moderate P efficiency | Liu et al., 2005 |
| 10 | 94-156 | High P efficiency | Zhang et al., 2009 |
| 11 | Ke feng 1 | High P efficiency | Li et al., 2005 |
| 12 | Jin dou 4 | Low P efficiency | Ding et al., 2006 |
| 13 | Da wu dou | Low P efficiency | Liu et al., 2005 |
| 14 | Hui min tie zhu gan | High P efficiency | Liu et al., 2005 |
