# Supplementary material for: The Acid Phosphatase-Encoding Gene GmACP1 Contributes to Soybean Tolerance to Low-Phosphorus Stress
Source: PLoS Genet. 2014 Jan 2;10(1):e1004061. doi: 10.1371/journal.pgen.1004061 (PMC3879153; doi:10.1371/journal.pgen.1004061)
Supplement: Table S3 — Summary of the 192 soybean accessions used. (DOCX) [file pgen.1004061.s010.docx]

Table S3 Summary of 192 soybean accessions

| **Taxa** | **Name** | **Origins** |
| --- | --- | --- |
| Nau1 | Nannong86-4 | Jiangsu |
| Nau2 | Suxie1 | Jiangsu |
| Nau3 | Bogao | Jiangsu |
| Nau4 | Nannong 94-156 | Jiangsu |
| Nau5 | Nannong1138-2 | Jiangsu |
| Nau6 | Kefeng 1 | Beijing |
| Nau7 | Guchengyishuhou | Hubei |
| Nau8 | Hefeng34 | Liaoning |
| Nau9 | Fengjiao66-12 | Liaoning |
| Nau10 | Liaodou11 | Liaoning |
| Nau11 | Yudou16 | Henan |
| Nau12 | Luhuangdou | Shanxi |
| Nau13 | Nanchengzeibuyao | Jiangxi |
| Nau14 | Shanghaibayuebai | Shanghai |
| Nau15 | Jurongdabiandou | Jiangxi |
| Nau16 | Fengxilouzihuang | Shandong |
| Nau17 | Yunyizao | Jiangsu |
| Nau18 | Xiayitaipingzihuadou | Henan |
| Nau19 | Suipingxiaoziyao | Henan |
| Nau20 | Wanxianbaidongdou | Chongqing |
| Nau21 | Dawudou | Jilin |
| Nau22 | Guiximayi | Guizhou |
| Nau23 | Jindou4 | Shanxi |
| Nau24 | Shangqiu73 | Henan |
| Nau25 | Xipinghesedou | Henan |
| Nau26 | Guangshanwenzhutian | Henan |
| Nau27 | Enshizaohuangdou | Hubei |
| Nau28 | Qingyuanxiaoqingdou | Guangdong |
| Nau29 | Huangdou | Jiangsu |
| Nau30 | Lishuizhongzihuangdou | Jiangsu |
| Nau31 | Jinhuadadou | Zhejiang |
| Nau32 | Yueyangliuyuebao | Hunan |
| Nau33 | Taishunqiudou | Zhejiang |
| Nau34 | Chundou | Fujian |
| Nau35 | Luanchuanchengguanxiaodou | Henan |
| Nau36 | Zhenfengliuyuehuang | Guizhou |
| Nau37 | Heidadou | Heilongjiang |
| Nau38 | Chadou | Shandong |
| Nau39 | Tongluniuchidou | Zhejiang |
| Nau40 | Deqingxiangzhudou | Zhejiang |
| Nau41 | Yangdali | Liaoning |
| Nau42 | Qingyujinhuang | Jiangxi |
| Nau43 | Wandouzao | Hubei |
| Nau44 | Heidou1 | Liaoning |
| Nau45 | Guangzehuangjiadou | Fujian |
| Nau46 | Zhenjianghuangdou | Jiangsu |
| Nau47 | Fengxiansuidaohuang | Shanghai |
| Nau48 | Xiaolidou | Liaoning |
| Nau49 | Shizhuzhuyaozi | Chongqing |
| Nau50 | Ganbusi | Jiangxi |
| Nau51 | Jisichanglidou | Heilongjiang |
| Nau52 | Yushanbayuebao | Jiangxi |
| Nau53 | Jiyuanshangzhuangshui | Henan |
| Nau54 | Heiyaodou | Heilongjiang |
| Nau55 | Mayidan | Shanxi |
| Nau56 | Longyanqiuwudou | Fujian |
| Nau57 | Juyexiaotuyan | Shandong |
| Nau58 | Xuchangbaihuacao | Henan |
| Nau59 | Kaifengguozhuangqing | Henan |
| Nau60 | Baihuamoshidou | Jilin |
| Nau61 | Nantongdayangqing | Jiangsu |
| Nau62 | Yushanqingdou | Jiangxi |
| Nau63 | Jinlongheidou | Guangxi |
| Nau64 | Xutongdadou | Jiangsu |
| Nau65 | Anluxiaohuangdou | Hubei |
| Nau66 | Huangpixiaoqingdou | Anhui |
| Nau67 | Tongshanbopihuangdoujia | Hubei |
| Nau68 | Wenfeng5 | Liaoning |
| Nau69 | Baimodou | Neimenggu |
| Nau70 | Duchangnidou | Jiangxi |
| Nau71 | Donganheidou | Guangxi |
| Nau72 | Mancangjin | Liaoning |
| Nau73 | Xingyanghuihuangdou | Henan |
| Nau74 | Xiaodongdou | Shichun |
| Nau75 | Fuyang170 | Anhui |
| Nau76 | Wujiangqingdou3 | Jiangsu |
| Nau77 | Banyemaodou | Jiangsu |
| Nau78 | Hongpiyehuangdou | Hubei |
| Nau79 | Gantai | Anhui |
| Nau80 | Nannong89-30 | Jiangsu |
| Nau81 | Jianliniumaohuang | Hubei |
| Nau82 | P171451 | America |
| Nau83 | Zhongdou14 | Beijing |
| Nau84 | Fujian341 | Fujian |
| Nau85 | Yantianqingpidou | Fujian |
| Nau86 | Anxianghuangdou | Hunan |
| Nau87 | Geyangqingdou | Jiangxi |
| Nau88 | York | America |
| Nau89 | Nannong73-93 | Jiangsu |
| Nau90 | Anshunbaijiaodou | Guizhou |
| Nau91 | 89-29 | Jiangsu |
| Nau92 | Guanyunhongmaoyou | Jiangsu |
| Nau93 | Donghaixiaoheidou | Jiangsu |
| Nau94 | Huaiyangqiuheidou | Jiangsu |
| Nau95 | Xiqingdou | Yunnan |
| Nau96 | Jiangyinheidou | Jiangsu |
| Nau97 | Donghaipingdinghuang | Shanghai |
| Nau98 | Lichengxiaolidou | Shandong |
| Nau99 | Hezepingdingdou | Shandong |
| Nau100 | Shuangjiangzongpidou | Yunnan |
| Nau101 | Gaoyaosanyuehuang | Guangdong |
| Nau102 | Kenjian2 | Heilongjiang |
| Nau103 | Dawuliuyuebao | Hubei |
| Nau104 | Guangfengmaliaodou | Jiangxi |
| Nau105 | Laoshuyan | Shanxi |
| Nau106 | Erwangdou | Jiangxi |
| Nau107 | Qujianghuangkengdong | Guangdong |
| Nau108 | Tianeliuyuehuang | Guangxi |
| Nau109 | Mayidou | Jiangxi |
| Nau110 | Chaqiushidou | Neimenggu |
| Nau111 | Liuhexiaoyeqing | Jiangsu |
| Nau112 | Yixianheidou | Hebei |
| Nau113 | Heibawangbian | Jilin |
| Nau114 | Zhijinsanjiaxiaodou | Hebei |
| Nau115 | Anyixiaoheidou | Shanxi |
| Nau116 | Dajinhuang | Heilongjiang |
| Nau117 | Jinxianzaochadou | Jiangxi |
| Nau118 | Maoyandou | Shanxi |
| Nau119 | Huimintiezhugan | Jiangsu |
| Nau120 | Jinzhoutangwang | Liaoning |
| Nau121 | Zhenbaxiaobaihuang | Shanxi |
| Nau122 | Tonganyouzaidou | Fujian |
| Nau123 | Binhaihongchadou | Jiangsu |
| Nau124 | Pingshanheidou | Hebei |
| Nau125 | Yushandawudou | Jiangxi |
| Nau126 | Anyiheidou | Shanxi |
| Nau127 | Jingdehuangdou | Anhui |
| Nau128 | Dajinyuan | Liaoning |
| Nau129 | Hongdouzi | Guizhou |
| Nau130 | Qiongshanxiawangwudou | Hainan |
| Nau131 | Qionghaixiaozhongwudou | Hainan |
| Nau132 | Haimenhonghuangdouyi | Hainan |
| Nau133 | Wuheidou2 | Shandong |
| Nau134 | Bamajiuyuehuang | Guangxi |
| Nau135 | Zhongdou27 | Beijing |
| Nau136 | Chuxiu | Liaoning |
| Nau137 | Dandou12 | Liaoning |
| Nau138 | Nanhuaheipidou | Yunnan |
| Nau139 | Feixidahuangdou | Anhui |
| Nau140 | Jianshuixiaoheidou | Yunnan |
| Nau141 | Shangraoganbusi | Jiangxi |
| Nau142 | Y4B5375 | Jiangsu |
| Nau143 | Yaanxiaolizao | Shichun |
| Nau144 | Ningnanheizi | Jiangsu |
| Nau145 | Shangraowanqing | Jiangxi |
| Nau146 | Xiaoheidou | Hebei |
| Nau147 | Linheidou | Hebei |
| Nau148 | Yunhefengheidou | Zhejiang |
| Nau149 | Xiaolihuang | Jilin |
| Nau150 | Dajinhuang | Heilongjiang |
| Nau151 | Yangchunxiaoli | Guangdong |
| Nau152 | Hengshanhongdou | Hunan |
| Nau153 | Hengyangyaodou | Hunan |
| Nau154 | Chongmingtiegengdou | , Shanghai |
| Nau155 | Edou7 | Hubei |
| Nau156 | Gongdou10 | Beijing |
| Nau157 | Anshunbaijiaodou | Guizhou |
| Nau158 | Wukehuang | Jiangsu |
| Nau159 | Heikehuangdou | Jiangsu |
| Nau160 | Peixiandabaijiao | Jiangsu |
| Nau161 | Y4B51486 | Jiangsu |
| Nau162 | Huanglingshanzidou | Shanxi |
| Nau163 | Chunheidou | Hubei |
| Nau164 | Yangyanjinghuangdou | Shanxi |
| Nau165 | Linjiangheidou | Heilongjiang |
| Nau166 | Heimoshidou | Neimenggu |
| Nau167 | Huangdou | Gansu |
| Nau168 | Xuyongxiaobaishuidou | Shichun |
| Nau169 | Ningdoubayuehuang | Jiangxi |
| Nau170 | Yongxinliuyuehuang | Jiangxi |
| Nau171 | Longshanneiziqing | Hunan |
| Nau172 | Woyangheidou | Anhui |
| Nau173 | Yunmengliuyuehuaye | Anhui |
| Nau174 | Dalihuang | Jilin |
| Nau175 | Nannongcai5 | Jiangsu |
| Nau176 | Qiandou6 | Guizhou |
| Nau177 | Malidou | Zhejiang |
| Nau178 | Dabuhuangdou | Guangdong |
| Nau179 | Daheidou | Guangdong |
| Nau180 | Ninghaiwanhuangdou | Jiangsu |
| Nau181 | Nannong88-31 | Jiangsu |
| Nau182 | Edou2 | Hubei |
| Nau183 | Guangji | Jiangsu |
| Nau184 | 94001-1 | Henan |
| Nau185 | Zheng92029 | Henan |
| Nau186 | Shangraoheiyangdou | Jiangxi |
| Nau187 | Changyuanfantunxiaotiane | Henan |
| Nau188 | Runanpingdingdou | Henan |
| Nau189 | Jindou14 | Shanxi |
| Nau190 | Xipinghongmiandou | Henan |
| Nau191 | OC7934 | Brazil |
| Nau192 | Xinchangliuyuedou | Zhejiang |
